# Photosensitizer in lipid nanoparticle: a nano-scaled approach to antibacterial function

**DOI:** 10.1038/s41598-017-07444-w

**Published:** 2017-08-11

**Authors:** Bishakh Rout, Chi-Hsien Liu, Wei-Chi Wu

**Affiliations:** 1 Graduate Institute of Biochemical and Biomedical Engineering, Chang Gung University, 259, Wen-Hwa First Road, Kwei-Shan, Tao-Yuan 333 Taiwan; 2 Research Center for Chinese Herbal Medicine and Research Center for Food and Cosmetic Safety, College of Human Ecology, Chang Gung University of Science and Technology, 261, Wen-Hwa First Road, Taoyuan, Taiwan; 3 Department of Chemical Engineering, Ming Chi University of Technology, 84, Gung-Juan Road, New Taipei City, Taiwan; 4 Department of Ophthalmology, Chang Gung Memorial Hospital, 5, Fu-Hsing Street, Taoyuan, Taiwan; 5 College of Medicine, Chang Gung University, 259, Wen-Hwa First Road, Taoyuan, Taiwan

## Abstract

Photosensitization-based antimicrobial therapy (PAT) is an alternative therapy aimed at achieving bacterial inactivation. Researchers use various photosensitizers to achieve bacterial inactivation. However, the most widely used approach involves the use of photosensitizers dispersed in aqueous solution, which could limit the effectiveness of photodynamic inactivation. Therefore, the approaches to encapsulate the photosensitizer in appropriate vehicles can enhance the delivery of the photosensitizer. Herein, Toluidine Blue O (TBO) was the photosensitizer, and lipid nanoparticles were used for its encapsulation. The lipid nanoparticle-based delivery system has been tailor-made for decreasing the average size and viscosity and increasing the formulation stability as well as the wettability of skin. Usage of an appropriate vehicle will also increase the cellular uptake of the photosensitizer into the bacterial cells, leading to the damage on cell membrane and genomic DNA. Evidence of effectiveness of the developed PAT on planktonic bacteria and biofilms was examined by fluorescence microscopy and scanning electron microscopy. Lipid nanoparticles protected the photosensitizer from aggregation and made the application easy on the skin as indicated in data of size distribution and contact angle. The use of lipid nanoparticles for encapsulating TBO could enhance photosensitization-based antimicrobial therapy as compared to the aqueous media for delivering photosensitizers.

## Introduction

Photosensitization-based antimicrobial therapy (PAT) is an alternative treatment option that has gained attention in research conducted in the last few years^1^. This is because of the growing resistance of microorganisms against commonly used antimicrobials^2^. PAT is a technique which uses a photosensitizer and irradiates it with light of a specific wavelength to generate reactive oxygen species (ROS) for causing unspecific oxidative damage to various parts of bacterial cellular architecture^3, 4^. PAT is more effective than traditional antimicrobial tools, as evidence suggests that microorganisms cannot develop resistance to it^5^. Given the challenge of hospital-acquired infections, it is prudent that more resources be devoted to carrying out research on alternative treatments such as PAT. The compound responsible for killing microbes in PAT is, effectively, the photosensitizer^6^. Of the numerous options available, a cationic photosensitizer such as Toluidine Blue O (TBO) possesses some inherent advantages for PAT^7^. Cationic photosensitizers are more effective against a broad spectrum of microbes, including Gram-negative bacteria. In addition, the use of a cationic photosensitizer provides selectivity for uptake into microbial cells, if used for treating infections localized on human tissue^8^. This reduces the chances of damage inflicted on healthy host mammalian tissue while conducting PAT. TBO has already been used by various research groups to achieve microbial inactivation^9–11^. A common thread connecting this research is the use of an aqueous medium for the dispersion of TBO. In addition to TBO, photosensitizers such as methylene blue, curcumin, and deuteroporphyrin have been applied to PAT while being dispersed in an aqueous medium^12, 13^. Such an approach is fraught with risks, including aggregation of the photosensitizer and its degradation, thereby limiting antibacterial inhibition^12, 14–16^. Prominent research groups involved in PAT have admitted that dispersing the photosensitizer in water is bound to result in aggregation^8^. Very little research exists about the use of nanocarrier-based approaches to enhance the efficiency of PAT.

In this study, we aimed to unveil the mechanisms responsible for enhancing the effectiveness of PAT after use of a nanocarrier for encapsulation. TBO was used as the photosensitizer, and lipid nanoparticles made from essential oil was chosen for encapsulation. As mentioned previously, the killing action of PAT obtained after irradiating the photosensitizer with light of a specific wavelength is due to the generation of ROS^4^. Various ROS species are generated after irradiation of the photosensitizer with light, and all have differing efficiencies in inactivating microorganisms^17, 18^. The mechanism by which the ROS are generated is, therefore, a key parameter in determining the microbial inactivation^19^. The rate of generation of these ROS is another key factor governing microbial inactivation. Since the generation of ROS depends on the levels of molecular oxygen, a quicker rate will deplete molecular oxygen levels and prevent effective microbial inactivation^20^. Finally, the proximity of generated ROS to the bacterial cellular architecture is another factor that determines the effectiveness of microbial inactivation^21^. The generated ROS have very short lifetimes and limited diffusion distances, and therefore a higher amount of cellular uptake will ensure that the generated ROS are physically able to reach cellular architecture and cause unspecific oxidative damage^22^. In this study, lipid nanoparticles were chosen as the delivery system to encapsulate the photosensitizer. In lipid nanoparticles, the aqueous phase and the organic phase are stabilized by a surfactant^23^. One phase is distributed in another in the form of small droplets. The surfactant helps in reducing the surface tension between the two phases. In addition, the use of a co-solvent helps in further reducing the interfacial tension^24^.

Nanotechnology-based approaches have been used in various science disciplines to achieve the enhancement of desirable effects. Recently, authors have used nano-scaled penicillin to enhance antibacterial inhibition against Gram-negative bacterial strains^25^. Silver nanoparticles have been a popular choice for achieving greater antibacterial inhibition in combination with antibiotics^26^. Researchers have also shown that the use of nano-fibers loaded with chlorhexidine and silver nanoparticles leads to heightened antibacterial activity^27^. Photosensitization-based antimicrobials have been under observation for a variety of uses, like topical use for anti-cancer applications^28^. Use of nanoparticle-based delivery systems designed using constituents such as surfactants has been shown to increase the effectiveness of antimicrobial activity^29^. Use of nanoparticle-based delivery systems has also been shown to exert enhanced anti-cancer action as compared to only photosensitizer formulations^30^. Usage of nanocarriers has also enabled dual use of photosensitizers as imaging agents, in addition to increasing their phototoxicity^31^. Usage of nanocarriers has also enabled a combined photothermal-photodynamic therapy with the usage of copper sulfide and chlorin e6^32^. Use of photosensitizers in the nanosized form has also shown improved delivery to tumors accompanied with increased photosensitization efficiency^33^. Therefore, the effect of encapsulation of TBO in a nanoparticle delivery system was investigated on the basis of the previously mentioned parameters governing the efficiency of PAT and compared with the more popular approach (i.e., TBO in an aqueous medium) used by researchers in the past. The effectiveness of photodynamic inactivation with TBO formulations was compared by using fluorescence microscopy with biofilm producing strains. Changes to biofilm and signs of damage were investigated after PAT by using scanning electron microscopy.

## Results and Discussion

### Lipid nanoparticle characterization

**Figure 1:**
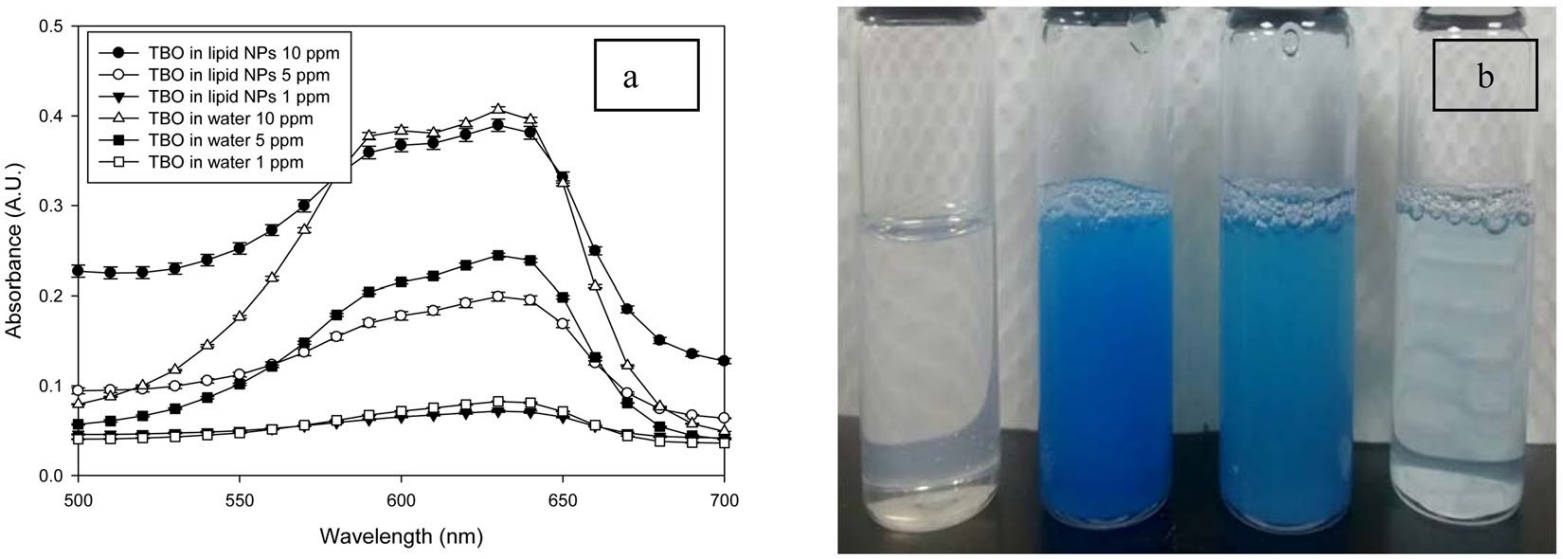
(**a**) UV-vis absorbance spectra of TBO in lipid nanoparticles formulation containing 1, 5, 10 ppm TBO. (**b**) Sample vials positioned from left to right are as follows: lipid nanoparticles formulation without TBO; TBO in lipid nanoparticles at a concentration of 10 ppm; 5 ppm and 1 ppm, respectively.

Use of the lipid nanoparticles resulted in improvement of many parameters of the TBO formulation (Table 1). The absorbance at the characteristic TBO peak of 626 nm increased correspondingly with the photosensitizer concentration (Fig. 1(a)). Absorbance also increased in regions other than the characteristic peak. The formulations can contribute to increased efficiency of PAT by absorbing more photons to excite the photosensitizer^34^. The lipid nanoparticles without any photosensitizer loading were isotropic and translucent. The formulation became increasingly darker with an increased concentration of TBO (Fig. 1(b)). After the use of lipid nanoparticles, the particle size decreased as compared to TBO in water. Additionally, the polydispersity index slightly improved and the zeta potential became negative. This resulted in the TBO in lipid nanoparticle formulation becoming more stable and uniformly sized. In aqueous solution, the photosensitizer was prone to aggregation and therefore attained bigger average particle sizes. The size distribution of TBO in water becomes non-uniform due to aggregation and instability. The observations have been clearly displayed in the TEM images of TBO in water and TBO in lipid nanoparticles (Fig. S1(a) and (b)). Among other parameters, the viscosity of formulation and its wettability on porcine skin suggested that TBO encapsulated in lipid nanoparticles was more capable of being applied on human skin. This was possibly due to the smaller contact angles of TBO in lipid nanoparticles on the porcine skin (Fig. S2(a) and (b)).

**Table 1:** Characterisation of TBO formulations.

Parameter	TBO in water	TBO in lipid nanoparticles
Average size (nm)	217.4 ± 6.8	122.3 ± 2.7
Polydispersity index	0.427 ± 0.413	0.478 ± 0.053
Zeta potential (mV)	6.533 ± 0.821	−0.333 ± 0.446
Viscosity (mPas)	92.78 ± 0.40	196.40 ± 0.40
Contact angle on glass (degree)	54.06 ± 5.55	19.47 ± 4.69
Contact angle on pig skin (degree)	52.69 ± 2.21	27.35 ± 3.75

The formulated lipid nanoparticles were able to achieve good encapsulation of the photosensitizer TBO. Encapsulation efficiency was found to be 83.01% by size exclusion chromatography experiment using Sephadex G-50 Superfine (Fig. S7). Storage tests were also carried out to find out the stability of the prepared lipid nanoparticles. Over a storage period of about 2 months, no significant changes in the average particle size (Fig. S8) or the polydispersity index of the formulation (Fig. S9) was noticed. This proved that the formulation of lipid nanoparticles was stable in encapsulating TBO over the said duration of two months. In addition, we examined the cell toxicity of different formulations on the suspended Chinese hamster ovary cells. The toxicity to the cells was decreased after the usage of lipid nanoparticles for encapsulating TBO. TBO in aqueous mode posed significant toxicity to the tested cells. TBO encapsulation in lipid nanoparticles somewhat mitigates the toxicity and reduces the toxic effect as indicated in Fig. S10.

### Factors affecting the efficiency of PAT on bacteria

**Figure 2:**
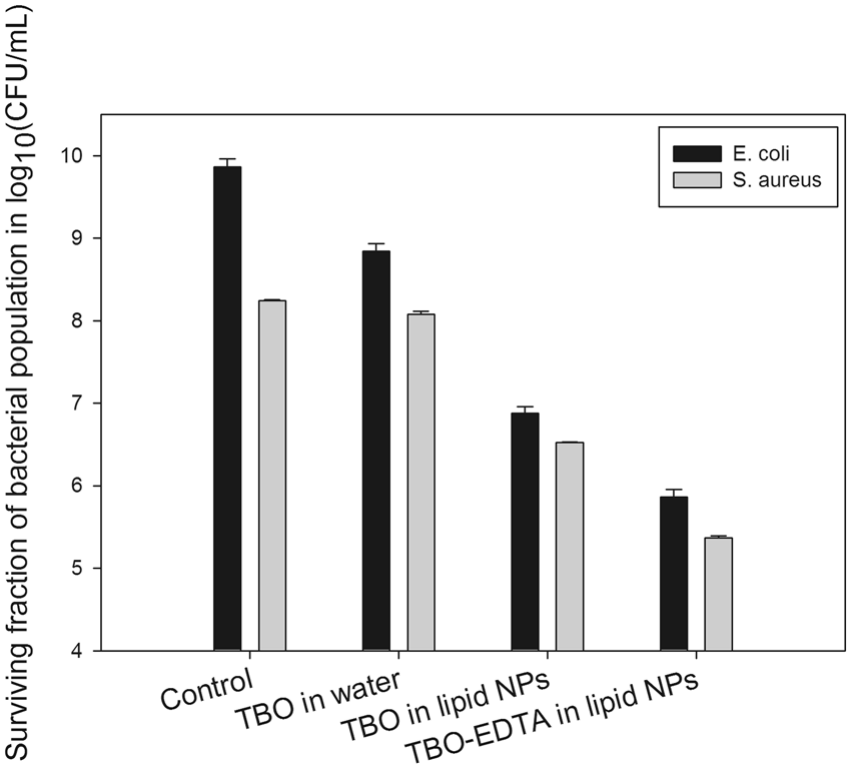
Effect of various formulations on inhibition of (a) *S*. *aureus* and (b) *E*. *coli* - Maximum inactivation was achieved with combination of TBO in lipid nanoparticles with EDTA. The inactivation of TBO in water was much lesser than TBO in lipid nanoparticles. Significant differences between the control groups and the treated groups have been denoted at a probability value < 0.05 (*).

In the case of both Gram-positive strain *S*. *aureus* (Fig. 2, gray bars) and Gram-negative strain *E*. *coli* (Fig. 2, black bars), maximum reduction in bacterial population after PAT was obtained by the formulation combining EDTA with TBO in a lipid nanoparticle delivery system (~3 logs for *S*. *aureus* and ~4 logs for *E*. *coli*). EDTA alone was not able to cause damage to both the bacterial strains (Fig. S3). Also, the lipid nanoparticles alone without TBO were not able to exert significant inhibitory effect on either of the bacterial strains (Fig. S3). The effect of TBO in water was less than for TBO encapsulated in lipid nanoparticles (Fig. 2). These results point to the fact that efficiency of PAT against microorganisms can be enhanced by use of a nanoparticle delivery system. The photosensitizer delivered in an aqueous solution was prone to aggregate and destabilize, as indicated in Fig. S1(a), and resulted in reduced PAT effectiveness. The choice of constituents in designing a nanoparticle delivery system is also important to achieving effective anti-microbial inactivation.

Essential oils have the ability to destabilize the lipid bilayer of the bacterial cell membranes, which allows foreign agents such as a photosensitizer to penetrate easily^35, 36^. Eucalyptus oil was chosen as the organic phase of the lipid nanoparticle delivery system for TBO in this study. Antibacterial inhibition was further enhanced by the synergistic effect of EDTA^37, 38^. EDTA chelates with the divalent cations (Ca^2+^, Mg^2+^) present in the membranes of bacterial cells and also causes destabilization of the membranes^39^. As a result, foreign agents can easily penetrate the bacterial cell. Such strategies can improve the antimicrobial efficiency of PAT against Gram-negative microorganisms by allowing the photosensitizer to penetrate easily. In a general trend, PAT can attain easy inactivation of Gram-positive organisms but faces problems during inactivation of Gram-negative organisms due to the thicker outer membrane of Gram-negative bacterial cells^40^. Such problems can be overcome by using a nanoparticle delivery system combined with penetration enhancers like EDTA to increase the antimicrobial effect of PAT on Gram-negative organisms.

### Analysis of damage to bacterial DNA by PAT

The nuclear material of any bacterial cell is important for many of the cell’s functions, including reproduction. Damage to the DNA of any bacterial cell is, therefore, a very effective way to reduce the microbial population. Conventional antimicrobials such as antibiotics are unable to cause effective damage to the nuclear material of bacterial cells, whereas PAT has been proven to cause DNA damage to even *Deinococcus radiodurans*, an organism that has one of the most efficient mechanisms of DNA repair^41^.

**Figure 3:**
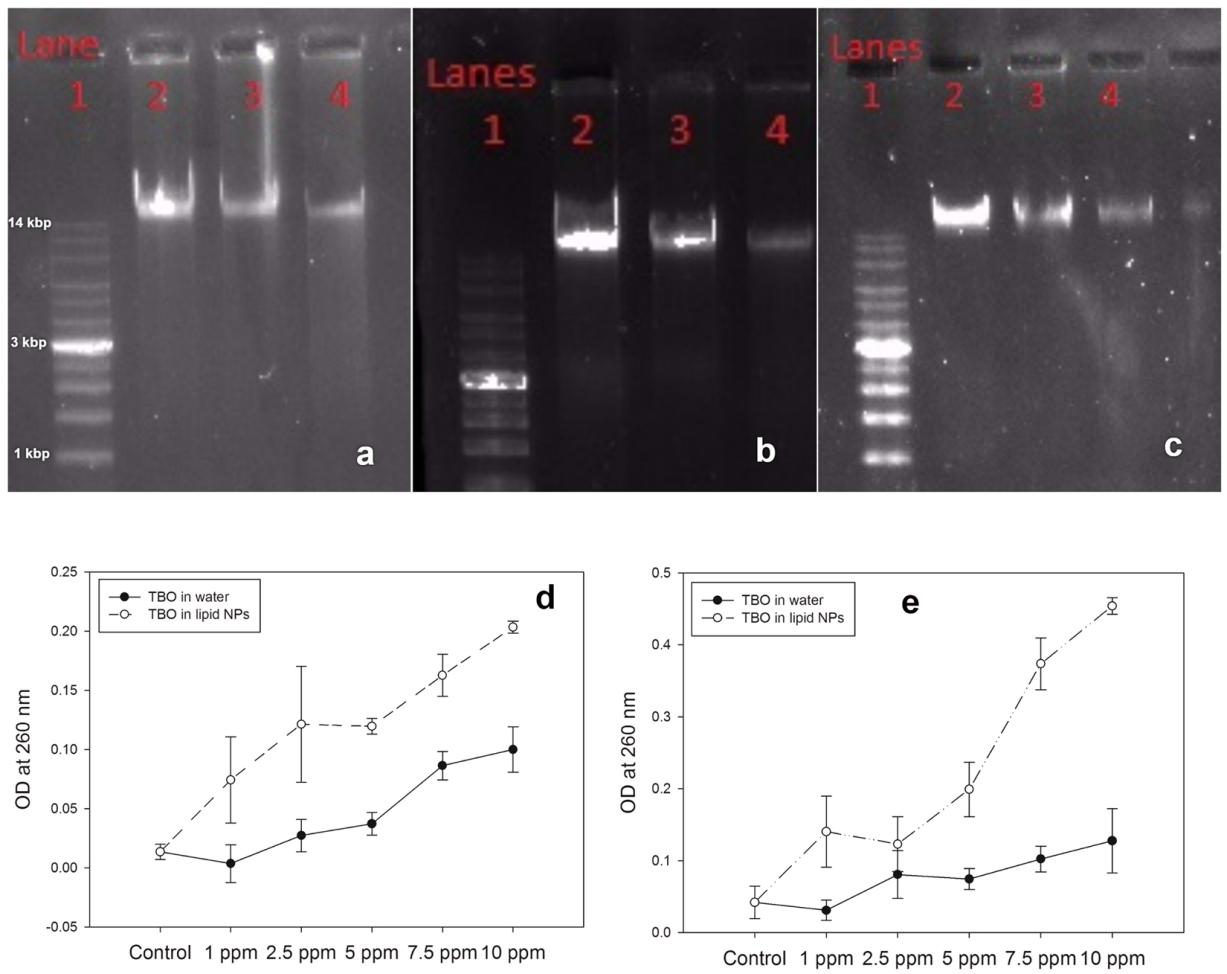
DNA damage assay by gel electrophoresis of (**a**) *E*. *coli*, (**b**) *P*. *aeruginosa* and (**c**) *S*. *aureus* after treatment with PAT using TBO in water and TBO in lipid nanoparticles. In all the images, lane 1 was loaded with DNA ladder. Lane 2 was loaded with DNA extracted from bacteria treated with sterile saline. Lanes 3 and 4 were loaded with DNA extracted from bacteria treated with PAT using TBO in water and TBO in lipid nanoparticles. Analysis of bacterial DNA exudation from (**d**) *E*. *coli* and (**e**) *S*. *aureus* after treatment with PAT using TBO in water and TBO in lipid nanoparticles revealed that DNA exudation was increased after usage of TBO in lipid nanoparticles.

Damage to DNA can be attributed to the ROS generated after irradiation of the photosensitizer with light. To augment PAT damage to DNA, the range of the ROS must be enhanced. Since DNA is located deep inside the cell and is protected by cell membranes, one way to increase damage is to increase cellular uptake of the photosensitizer. As a result, the photosensitizer will be closer to the cell’s DNA and can therefore inflict more damage. As shown in the following sections, use of a lipid nanoparticle-based delivery system enhanced the cellular uptake of the photosensitizer. Correspondingly, the damage to DNA after PAT was also increased in all three bacterial strains (Fig. 3). In each of the gel electrophoresis assays, the first lane is loaded with the DNA ladder. The second lane is loaded with the DNA extracted from the control group of bacterial samples that were treated with only sterile buffered saline. A high amount of DNA is present in the second lane in all three assays. When treated with PAT using TBO in water, there was a decrease in the amount of DNA in all three bacterial strains, as shown by Lane 3 in all of the gel electrophoresis assay images. However, maximum degradation of DNA occurred when the bacterial samples were treated with PAT using TBO in lipid nanoparticles (Fig. S4). This is denoted by Lane 4 in all of the gel electrophoresis assay images. These images proved that TBO encapsulated in lipid nanoparticles was able to cause more DNA damage than TBO in water. Hence, encapsulation in lipid nanoparticles is an effective way of increasing antimicrobial efficiency by PAT. The mode of damage and the observations are similar to those made by previous workers^42^. In their work, they witnessed that photodynamic action on a bacterial strain caused damage to the bacterial membrane as well the DNA contents.

**Figure 4:**
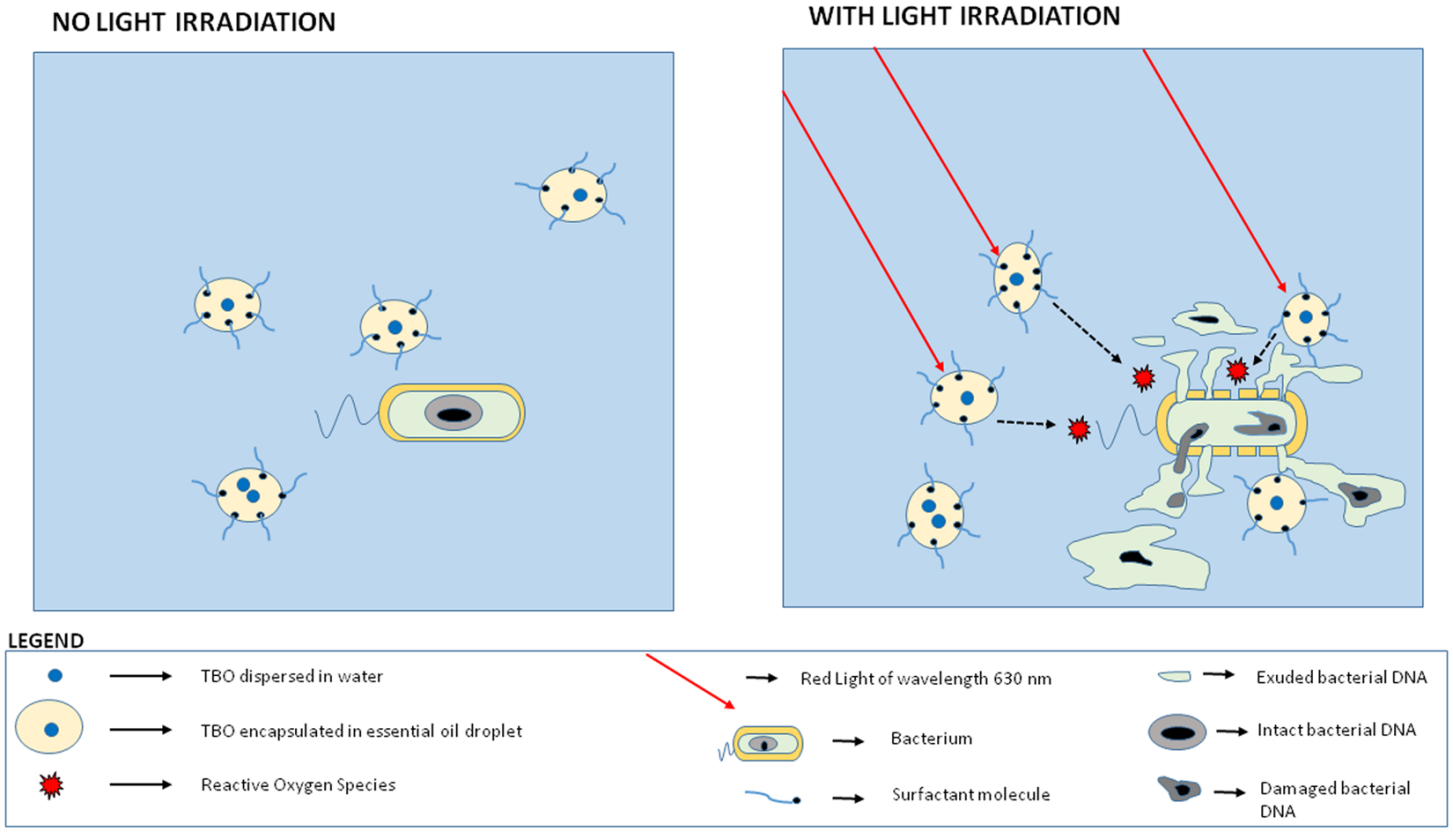
Schematic diagram for bacterial exudation studies after treatment with PAT. Usage of lipid nanoparticles for encapsulating TBO enhanced damage to bacterial cell and increased oxidative damage the leakage of cellular contents as compared to TBO in water.

Damaged DNA and other cytoplasmic material exuding from bacterial cells can be detected by spectrophotometric absorbance at 260 nm^43^. Since the cell membrane is a prime target for oxidative damage by ROS produced after light irradiation of the photosensitizer, it is expected that some material will exude from the cell after PAT^44, 45^. More damage to the cell membranes is expected to cause exudation of higher amounts of DNA and cytoplasmic material from the cell^46^. In our study, both strains showed an increase of exuded material (increased absorbance at 260 nm) in correspondence to increasing concentrations of photosensitizer, i.e., a prominent dose-dependent effect (Fig. 3(d) and (e)). In addition, the exudation was much higher when TBO was encapsulated in lipid nanoparticles, compared to only TBO in water. In the case of *S*. *aureus*, the exuded material was more than twice that in the case when the bacterial cells were treated with TBO in water. In the case of the control group of bacterial samples, the bacterial cells were treated with only sterile buffered saline and showed almost no exudation of cellular material. All results point to the effectiveness of a lipid nanoparticle-based delivery system in increasing cellular uptake of the photosensitizer, resulting in localized generation of ROS and thus enhanced oxidative damage to bacterial cellular structures, as described in Fig. 4. This is possible due to the use of lipid nanoparticles for encapsulation of TBO. Encapsulation decreases the size of TBO particles and prevents aggregation and instability. In addition, the choice of essential oil allows the formulation to destabilize the cell wall and enables a higher cellular uptake of the photosensitizer. Hence, the photosensitizer was closer to the bacterial cell and could therefore cause more oxidative damage to the cellular structure because of the closeness of ROS. Similar observations have been made by previous authors^47^. They concluded that damage to bacterial genomic and plasmid DNA occurs only after the membrane has been breached. Therefore, inclusion of agents like essential oil in our lipid nanoparticles will play the role of softening up the bacterial cell membrane so that more of reactive oxygen species can exert damage to the inner DNA contents.

### Morphology change evaluation via scanning electron microscopy and fluorescence microscopy

**Figure 5:**
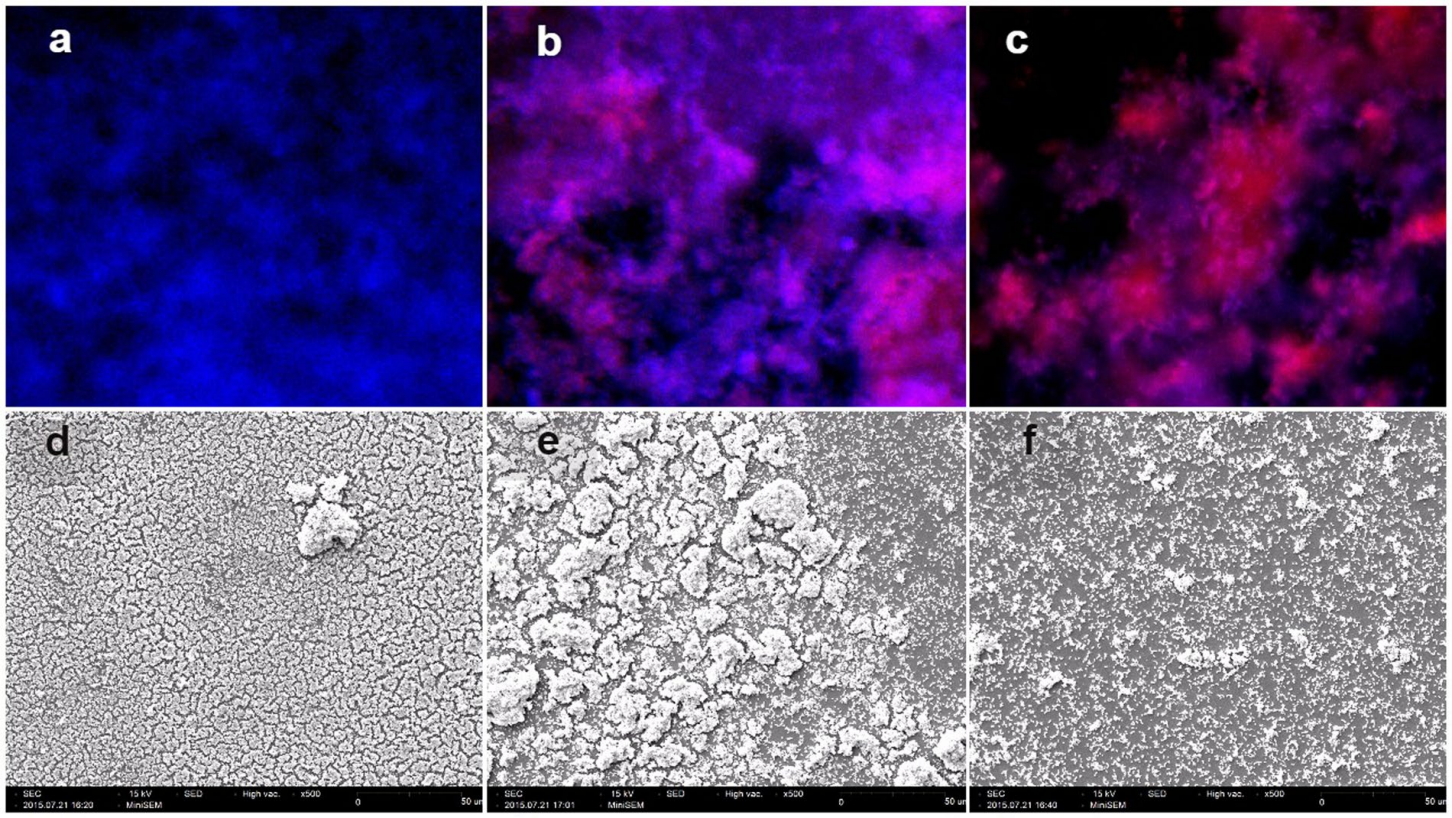
Fluorescence microscopy images of *S*. *aureus* SA 113 biofilms treated with PAT using TBO formulations showed that (**a**) control group biofilm treated with PBS had only live bacterial cells. (**b**) Bacterial biofilms treated with PAT using TBO in water had both live (blue) and dead (red) bacterial cells. (**c**) Majority of bacterial cells were dead after treatment with TBO in lipid nanoparticles. Scanning electron microscopy images (at 500X magnification) of *S*. *aureus* SA 113 biofilms treated with PAT using TBO formulations (**d**) Control group had dense and continuous biofilm structure. (**e**) Biofilms treated with PAT using TBO in water had some fragmentation. (**f**) Biofilms treated with PAT using TBO in lipid nanoparticles were intensely fragmented and damaged, and also reduced in population.

A drastic reduction in bacterial population was observed in the biofilm treated with PAT using TBO in lipid nanoparticles (Fig. 5(c)). An altered morphology was observed, as compared to the control group biofilm treated with only sterile buffered saline (Fig. 5(a)). While the control group biofilm was continuous, the biofilm treated with TBO in lipid nanoparticles was fragmented. This suggested probable damage caused to the bacterial biofilm from generated ROS after treatment with TBO in lipid nanoparticles using PAT. On the other hand, the bacterial biofilm treated with PAT using TBO in water was still largely intact, although showing some signs of fragmentation (Fig. 5(b)). These results indicated to the effectiveness of lipid nanoparticle-encapsulated TBO in inactivating microorganisms using PAT.

Hoechst dye is a fluorescent cell-permeable dye that binds itself to the DNA of bacterial cells. Upon binding, the stain can emit blue fluorescence after being excited with specific light (350 nm). Propidium iodide is another fluorescent dye that penetrates bacterial cells with damaged membranes and quenches the fluorescence of Hoechst dye. Propidium iodide emits red fluorescence after being excited with specific light (535 nm). Attained images suggested that a majority of the bacterial cells in the *S*. *aureus* SA 113 biofilms treated with TBO in lipid nanoparticles (Fig. 5(c)) were dead cells (red color). On the other hand, biofilms treated with TBO in water (Fig. 5(b)) contained both live (blue) and dead (red) cells. The control group of biofilm was treated with sterile buffered saline (Fig. 5(a)) and contained live cells only. This proves the effectiveness of lipid nanoparticle-encapsulated TBO in inactivating microbial populations by PAT.

### Analysis of photosensitizer uptake in bacterial cells

**Figure 6:**
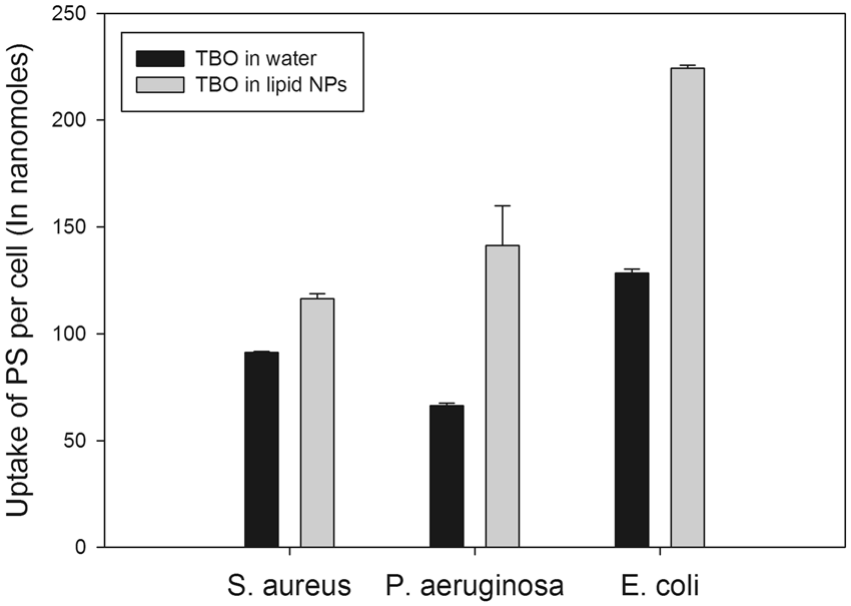
Uptake of photosensitizer molecules in bacterial cells of each type of strain expressed as the nano moles of photosensitizer per bacterial cell. Usage of lipid nanoparticles for encapsulating TBO enhanced the cellular uptake of photosensitizer as compared to usage of only TBO in water. Symbols like “*” indicate statistical significance between the groups of TBO in water and TBO in lipid nanoparticles; p < 0.05.

The damaging action of PAT is due to the ROS generated after irradiation of the photosensitizer with light of a specific wavelength. While these ROS can be quite reactive, resulting in the oxidation of any molecule in proximity, they have many limitations. They are relatively unstable and have small diffusion distances^22^. Therefore, they need to be generated as closely to the bacterial cell as possible to ensure effective damage. Thus, enhancing the uptake of the photosensitizer is key in increasing the damage caused by antimicrobial PAT^21^. Standard curves of absorbance of TBO in water and TBO in lipid nanoparticles were linear and had good correlation coefficients (Fig. S5(a) and (b)). These were used to calculate cellular uptake of the photosensitizer after PAT. The use of lipid nanoparticles as a delivery system increased the cellular uptake of the photosensitizer in all three bacterial strains, as compared to TBO in water (Fig. 6). This was possible due to the careful selection of the constituents of the lipid nanoparticles. Using an essential oil as the organic phase resulted in the destabilization of the bacterial cell membrane^35, 36^. As a result, the encapsulated photosensitizer was more effective at penetrating the bacterial cell. Further, the use of lipid nanoparticles as a nanoparticle delivery system allowed the photosensitizer to remain stable without aggregation. In the case of TBO in water, aggregation and instability caused the photosensitizer to reach both larger average sizes and non-uniform sizes (Fig. S1(a)). However, TBO in lipid nanoparticles with a slightly negative zeta potential could prevent TBO aggregation (Table 1, Fig. S1(b)). It also allowed a much smaller average particle size. When combined, these factors resulted in better penetration of the photosensitizer into the bacterial cell when encapsulated by lipid nanoparticles.

### Dependence of ROS generation during PAT on the type of formulation

It is well known that the damaging action of PAT on microorganisms is due to the ROS generated after irradiation of the photosensitizer with light of a suitable wavelength. Some examples of these ROS are singlet oxygen (^1^O_2_), hydroxyl radical (^.^OH), and superoxide anion (O^2−^). Generation of ROS can occur by two mechanisms, popularly known as Type I and Type II mechanisms. Type I mechanism leads to hydroxyl radicals and superoxide anions, whereas Type II mechanism produces singlet oxygen. Combining PAT with known quenchers of ROS is a possible means of determining the mechanism of photosensitization of the photosensitizer, and has been used in previous research^19^.

**Figure 7:**
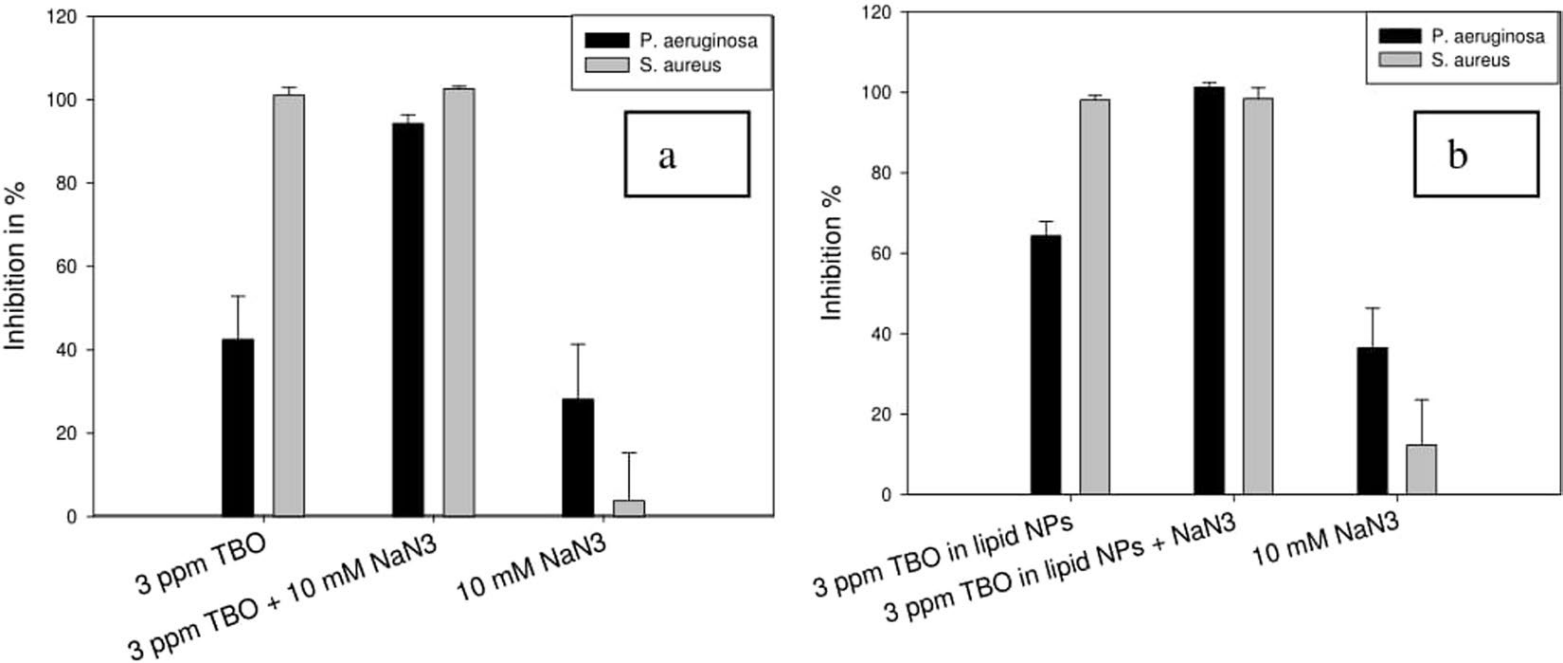
Effect of sodium azide combined with (**a**) TBO in water and (**b**) TBO in lipid nanoparticles on inhibition of *P*. *aeruginosa* and *S*. *aureus* - Inhibition increased in both the cases after combination with sodium azide.

Sodium azide is a specific quencher of singlet oxygen^48^. In previous work, researchers have noted that sodium azide has no inhibitory effect on the growth of bacteria, even when used at a concentration of 50 mM. In our studies, we observed a contradictory effect. Used at a concentration of 10 mM, sodium azide posed some inhibitory effect on the growth of *P*. *aeruginosa* and *S*. *aureus* strains used in our study (Fig. 7(a) and (b)). The inhibition of both *P*. *aeruginosa* (Fig. 7(a), black bars) and *S*. *aureus* (Fig. 7(a), gray bars) increased when TBO in water was combined with sodium azide. Previous research suggests that such an increase in inhibition is due to formation of azidyl radicals after reaction between hydroxyl radicals (produced due to light irradiation of TBO in water) and azide radicals^48^, which means that the process of photosensitization is Type I mechanism producing hydroxyl radicals. However, in our observations, it was difficult to confirm that point since sodium azide itself posed some toxicity to the bacteria. The same observations were made when TBO in lipid nanoparticles was combined with sodium azide. Inhibition of *P*. *aeruginosa* increased after the combination of sodium azide with TBO in lipid nanoparticles (Fig. 7(b), black bars). In the case of *S*. *aureus*, the inhibition levels were near complete inhibition and remained unchanged after the addition of sodium azide (Fig. 7(b), gray bars). In both the cases, we were unable to predict the effect since sodium azide itself has some toxicity. Therefore, in our studies, we also used L-histidine as a singlet oxygen quencher^49^.

**Figure 8:**
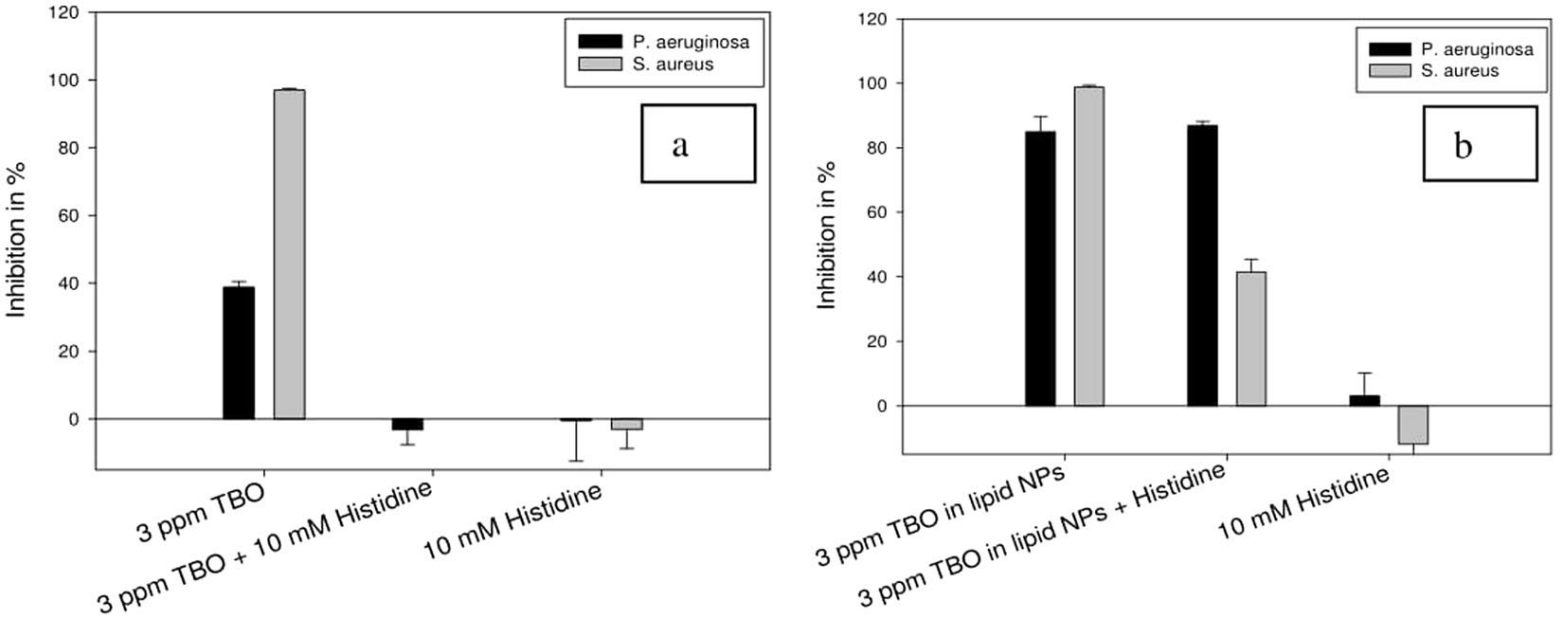
Effect of L-histidine combined with (**a**) TBO in water and (**b**) TBO in lipid nanoparticles on inhibition of *P*. *aeruginosa* and *S*. *aureus* - Inhibition was reduced to almost nil in the cases of both the strains of bacteria when TBO in water was combined with L-histidine. However, inhibition of *S*. *aureus* showed a decrease when TBO in lipid nanoparticles was combined with L-histidine. On the other hand, inhibition of *P*. *aeruginosa* remained unchanged after combination of TBO in lipid nanoparticles with L-histidine.

Unlike sodium azide, L-histidine itself had no inhibitory effect on the growth of either bacterial strain used in our study (Fig. 8(a) and (b)). When L-histidine was combined with TBO in water for PAT, the inhibitory action on both *P*. *aeruginosa* (Fig. 8(a), black bars) and *S*. *aureus* (Fig. 8(a), gray bars) was nullified. L-histidine itself had no effect, whether inhibitive or promotive, on the growth of either bacterial strain. This suggested the production of singlet oxygen from TBO in water by Type II mechanism of photosensitization, and subsequent quenching due to the addition of L-histidine. Quenching by L-histidine left behind almost no singlet oxygen to exert unspecific oxidative damage to bacterial cellular architecture, and therefore bacterial inhibition was nullified.

When L-histidine was combined with TBO in lipid nanoparticles, there was no change in the inhibition caused to *P*. *aeruginosa* (Fig. 8(b), black bars). There was, however, a marked decrease in the inhibition caused to *S*. *aureus* (Fig. 8(b), gray bars). This suggests that both singlet oxygen and hydroxyl radicals are produced from TBO in lipid nanoparticles after irradiation of light. Though L-histidine quenched the singlet oxygen, hydroxyl radical remained for the killing action. These hydroxyl radicals are much more toxic against a Gram-negative bacterium like *P*. *aeruginosa*, but are not effective against a Gram-positive bacterium lie *S*. *aureus*. Therefore, we observed almost complete inhibition in the case of *P*. *aeruginosa*, but less inhibition in the case of *S*. *aureus*. The observation regarding the higher toxicity of hydroxyl radicals to Gram-negative bacteria as compared to singlet oxygen was made by previous researchers^19^. Therefore, increased production of hydroxyl radicals by a predominantly Type I mechanism after encapsulation of TBO in lipid nanoparticles is a desirable effect. As they cannot be easily inactivated by bacterial virulence factors, hydroxyl radicals are more toxic to bacterial cells^18^. Singlet oxygen, on the other hand, can be inactivated by bacterial virulence factors^17^. Also, as mentioned earlier in the text, hydroxyl radicals are more toxic to Gram-negative bacterial species than to other ROS, such as singlet oxygen^19^. Thus, encapsulation in lipid nanoparticles can help in the effective inactivation of bacterial species, especially Gram-negative bacterial species.

Studies were also carried out using L-methionine, a known scavenger of hydroxyl radicals^50^. However, the data has been shown in only the supplementary section and was not included in the main manuscript to limit its size. When combined with TBO in water, there was a slight decrease in inhibition of both the bacterial strains (Fig. S11(a,b)). This suggests that some amount of hydroxyl radicals are also produced by TBO in water via Type II mechanism. This is apt, since no PS undergoes strictly one process of photosensitisation, and there exists a balance between both the two types of photosensitisation mechanisms. In the case of PDT with encapsulated TBO, there was also decrease in inhibition. However, complete protection from killing was not seen. This is because encapsulated TBO undergoes photosensitisation by both mechanisms as shown in the experiments using L-histidine. Therefore, L-methionine scavenged the hydroxyl radicals generated from encapsulated TBO, but the remainder of singlet oxygen was responsible for the kill (Fig. S12(a,b)). In addition, it is also to be noted that the drop in inhibition was higher in case of P. aeruginosa as compared to the drop observed in the inhibition levels of S. aureus. This can be attributed to the susceptibility of S. aureus to singlet oxygen. Gram-positive bacteria are more susceptible to singlet oxygen as compared to hydroxyl radicals^19^. The overall results elucidated that TBO in water started photosensitisation primarily via Type II mechanism of singlet oxygen generation. On the other hand, TBO in lipid nanoparticles undergoes photosensitisation by a combination of Type I and Type II mechanisms, giving rise to both singlet oxygen as well as hydroxyl radicals. This can be advantageous in killing Gram-negative pathogens such as *P*. *aeruginosa* and *E*. *coli*. One of the challenges that contemporary PDT has faced till now is that it has not been able to ensure significant inactivation of Gram-negative microorganisms as compared to Gram-positive organisms.

### Qualitative comparison of singlet oxygen generated from photosensitizer formulations

**Figure 9:**
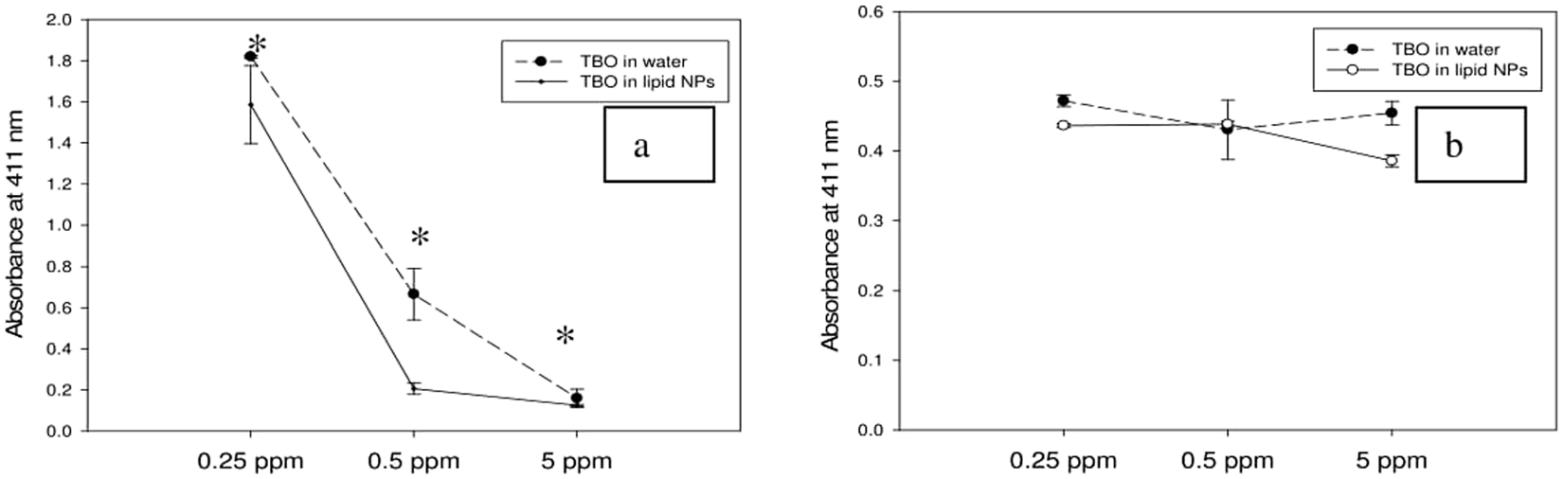
1,3-DPBF levels in solution after (**a**) light irradiation of TBO in water and TBO in lipid nanoparticles and (**b**) incubation of 1,3-DPBF with TBO in water and TBO in lipid nanoparticles in dark - Lower levels of 1,3-DPBF indicate the generation of a higher amount of singlet oxygen. Encapsulation in lipid nanoparticles led to generation of higher amount of singlet oxygen as compared to TBO in water. Symbols like “*” indicate statistical significance between the groups of TBO in water and TBO in lipid nanoparticles; p < 0.05.

We chose to monitor the production of singlet oxygen as a model species that causes damage to bacterial cells. Even though singlet oxygen isn’t the only species generated by the photosensitizer after light irradiation, it is nevertheless important and can be used to monitor the changes in ROS production after encapsulation. The monitoring of singlet oxygen was chosen by employing the spectrophotometric detection of 1,3-DPBF as a singlet oxygen-specific trap. A lower level of 1,3-DPBF suggests a higher generation of singlet oxygen, and therefore possible higher inactivation of microorganisms. Previous authors have used 1,3-DPBF to study the production of singlet oxygen after light irradiation of various photosensitizers^51^. It was observed that after encapsulation in lipid nanoparticles, a slightly higher amount of singlet oxygen was produced after light irradiation (Fig. 9(a)). The same observation was made at three different concentrations of photosensitizer. In the case of incubation of the photosensitizer in dark, no change was observed in the levels of 1,3-DPBF (Fig. 9(b)). This observation was the same across all concentration ranges of both TBO in water and TBO in lipid nanoparticles. This suggests that singlet oxygen was produced only after irradiation of the photosensitizer with light, and was not produced in dark conditions. Hence, encapsulation in lipid nanoparticles might help in increasing the amount of ROS generated after light irradiation, and therefore ensure more effective anti-microbial inactivation.

We chose 1,3-DPBF as a singlet oxygen-specific trap to monitor the kinetics of singlet oxygen generation. This was achieved by monitoring the spectrophotometric absorbance at 411 nm during different time intervals of light irradiation of the photosensitizer. The decreased concentration of 1,3-DPBF was used as an indirect measure of the production of singlet oxygen. This method was quite similar to reported methods in which the decay in fluorescence of 1,3-DPBF is used to estimate the rate of generation of singlet oxygen^52^. Following this, the concentration of 1,3-DPBF was plotted versus time. However, such plotting of concentration of 1,3-DPBF versus time produced linear plots in only very low concentrations (Fig. S4(a)). In other cases, the logarithm of the concentration (or the inverse of concentration) of 1,3-DPBF versus time produced plots that indicated the best correlation (Fig. S6(b–d)). These plots were used to predict the kinetics followed by the generation of singlet oxygen after irradiation of light. At very low concentrations of photosensitizer, the generation of singlet oxygen followed first-order kinetics (Table 2 and Table 3). However, as the concentration of photosensitizer was increased, the generation of singlet oxygen began following second-order kinetics (Table 2 and Table 3).

**Table 2:** Kinetics governing the generation of singlet oxygen from TBO in water*.

TBO in water	Rate Law	Rate constant	Correlation coefficient (R^2^)
5 ppm	Rate = 0.019000 × C_TBO_	0.01900 s^−1^	0.9091
10 ppm	Rate = 0.04385 × C_TBO_^2^	0.04385 M^−1^s^−1^	0.8939
25 ppm	Rate = 0.03385 × C_TBO_^2^	0.03385 M^−1^s^−1^	0.8075

*C_TBO_: Concentration of TBO.

**Table 3:** Kinetics governing the generation of singlet oxygen from TBO in lipid nanoparticles*.

TBO in lipid nanoparticles	Rate Law	Rate constant	Correlation coefficient (R^2^)
5 ppm	Rate = 0.01670 × C_TBO_	0.01670 s^−1^	0.9096
10 ppm	Rate = 0.03285 × C_TBO_^2^	0.03285 M^−1^S^−1^	0.9064
25 ppm	Rate = 0.01855 × C_TBO_^2^	0.01855 M^−1^S^−1^	0.8581

^*^C_TBO_: Concentration of TBO.

**Figure 10:**
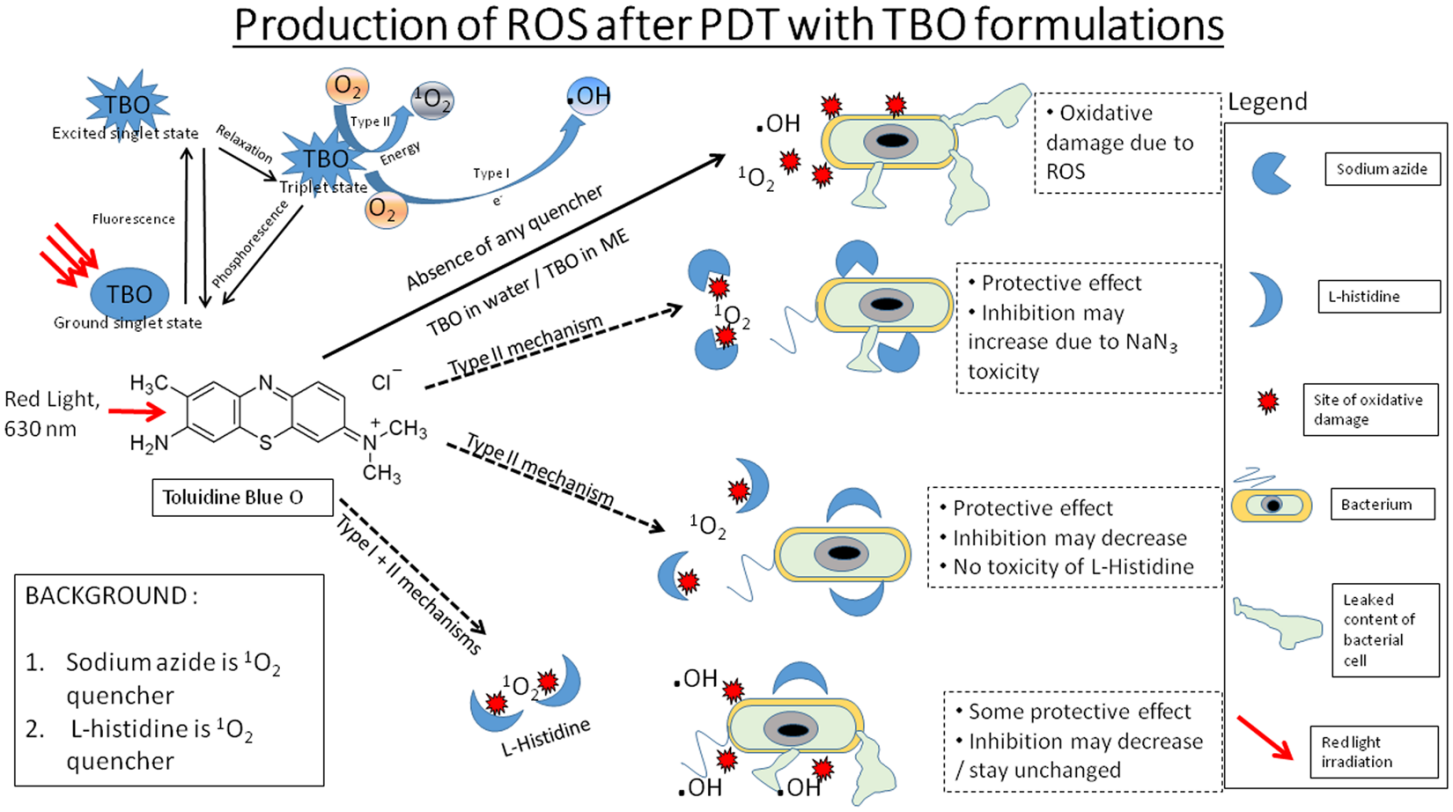
Antimicrobial mechanisms proposed for TBO in lipid nanoparticles. With the use of specific quenchers, it was proven that use of lipid nanoparticles for TBO encapsulation changes the mechanism of photosensitization from predominantly Type II to a combination of Type I and Type II mechanisms and therefore increases the amount of hydroxyl radicals produced to increase bacterial inactivation due to PAT.

Nevertheless, in all cases, one trend was clearly visible. The rate of generation of singlet oxygen following light irradiation, as shown by the rate constant, was slower after encapsulation of TBO in lipid nanoparticles (Table 3). TBO in water, at any concentration, led to the generation of singlet oxygen at a much faster rate (Table 2). This is desirable since the production of any kind of ROS depends on the levels of molecular oxygen^4^. A very fast rate of generation of singlet oxygen will deplete molecular oxygen levels more quickly, and the production of ROS may stop afterward. This is of more importance in treating deep tissue infections, where it is very difficult to replenish molecular oxygen levels. Studies by previous authors have also confirmed that a slower rate of generation of singlet oxygen is extremely desirable in PAT^20^. This can be achieved by either cycling the light irradiation through cycles of switching on-switching off-switching on, or by encapsulation in a nanoparticle delivery system, as done in this study. In addition, encapsulation in lipid nanoparticles improved other key parameters of PAT. As described in Fig. 10, use of quenchers with the photosensitizer enabled the determination of ROS involved in PAT with TBO formulations. There was a change of photosensitization mechanism from a predominantly Type II-based mechanism in PAT with TBO in water to a combination of Type I and Type II mechanisms in PAT with TBO in lipid nanoparticles. Such a change promoted the generation of more toxic hydroxyl radicals, which cannot be inactivated by bacterial virulence factors. Further, hydroxyl radicals are more effective than other ROS against Gram-negative strains. The use of lipid nanoparticles also improved the cellular uptake of the photosensitizer, which allowed the photosensitizer to generate ROS in closer proximity to the vital structures of the bacterial cell (such as DNA) and therefore caused effective inactivation of bacteria. The third key parameter of PAT was the rate of generation of singlet oxygen, which was slowed by the use of lipid nanoparticles as a delivery system. This prevented the quick depletion of molecular oxygen levels, and thus provided the chance for more effective inactivation of bacteria using PAT.

## Conclusion

This study provides more evidence toward increasing the effectiveness of antimicrobial PAT by the use of a nanoparticle system for delivering the photosensitizer. While the general method of operation in PAT-based research has been to use the photosensitizer in aqueous media, such an approach has many disadvantages that could result in decreased antimicrobial effectiveness of PAT. A lipid nanoparticle-based delivery system is tailor-made to overcome such challenges by decreasing the average particle size and preventing aggregation. This increased stability is one of the factors behind more effective inactivation of microorganisms using PAT. Use of lipid nanoparticles also improved some desirable properties, such as viscosity and wettability of porcine skin, allowing it to be better used as a photosensitizer delivery agent. Choice of a lipid nanoparticle system for delivering TBO increased the uptake of the photosensitizer TBO into the bacterial cells, and resulted in higher inactivation of bacteria. Increased uptake of the photosensitizer into bacterial cells caused higher amounts of DNA damage. The delivery system also provided a sustained generation of singlet oxygen as compared to the mode of photosensitizer in aqueous media. Evidence of effectiveness of PAT using lipid nanoparticles for encapsulating TBO was shown in the fluorescence microscopy images. The majority of cells treated with PAT using TBO in lipid nanoparticles were dead. Similar observations were found from the scanning electron microscopy images, with greater signs of damage to the biofilm occurring after treatment with PAT using TBO in lipid nanoparticles. Usage of a lipid nanoparticle based delivery system resulted in greater killing of bacteria as compared to the killing when only photosensitizer in aqueous media was used. Therefore, this study shows a way to increase the effectiveness of antimicrobial PAT using lipid nanoparticles as a nanoparticle delivery system for a photosensitizer such as TBO.

## Methods

### Bacterial Strains

*P*. *aeruginosa* (ATCC 27853), *E*. *coli* (ATCC 25922) and *S*. *aureus* (ATCC 6538) were obtained from Bioresource Collection and Research Center, Taiwan. Some experiments used biofilm-producing *S*. *aureus* SA 113 (ATCC 35556). Colonies were maintained on agar supplemented with the necessary medium, and transferred to fresh agar plates every two weeks. Cultures were grown by taking 2–3 colonies from agar plate to 25 mL of medium in a flask (3 mL of in tubes for some experiments) and maintaining at 37 °C at 180 rpm for 12–16 hours. Medium used for *P*. *aeruginosa* and *S*. *aureus* was Difco^TM^ nutrient broth. Difco^TM^ tryptic soy broth was for *E*. *coli*. On the other hand, *S*. *aureus* SA 113 required tryptic soy broth supplemented with 0.5% glucose.

### Photosensitizer formulations

Distilled water, eucalyptus oil (Sigma Aldrich), glycerol (J.T. Baker) and Tween80 (Croda) were used as the constituents for preparing stock lipid nanoparticles containing 1000 ppm TBO (Sigma Aldrich). The lipid nanoparticles were prepared using an ultrasonic cell disruptor (Microson Misonix) and a vortex (Genie2, Scientific Industries). When used for PAT, the stock was appropriately diluted in distilled water to obtain the desired concentration. In some experiments, ethylenediaminetetraacetic acid (EDTA) was added in appropriate concentrations to the lipid nanoparticle formulations before using in PAT experiments. The stock was prepared daily, and older stocks were discarded. Distilled water was obtained using an A10 MilliQ (Millipore, Molsheim, France), with a molar resistivity of 18.2 Mega ohms.

### Lipid nanoparticles characterization studies

The stock lipid nanoparticle was prepared daily. The characterization of the particle size, size distribution and the zeta potential was done with the help of a ZetaSizer® Nano ZS 90 (Malvern Instruments, Worcestershire, UK) at a backscatter angle of 173° and a temperature of 25 °C. Samples were diluted with water to a suitable concentration before the analysis. The refractive index for unloaded and TBO-loaded in lipid nanoparticles was set at 1.590. Twenty measurements were carried out for each sample. Turbidity of the TBO formulations was measured using a Hach 2100 N turbidimeter. Viscosity measurements were taken with a Brookfield DV-II+ Pro viscometer connected to proprietary Rheocalc™ software. For measuring contact angles, Phoenix Mini contact angle analyser was used with proprietary Surfaceware™ software. For measuring UV-vis absorbance spectrum, 100 µL aliquots of appropriate concentrations were loaded to the wells of a 96-well plate and absorbance monitored in the range of 500–700 nm using a microplate reader (Synergy, Biotek, Hong Kong).

### Photosensitization-based antimicrobial activity studies

The inactivation studies using 3 M Petrifilm™ aerobic growth count plates were carried out according to a protocol described in another research^53^. The PAT experiments used an LED light (Simon-Tech Inc., Taiwan). At a distance of 20 cm for an irradiation duration of 15 minutes, the light source was able to deliver total energy of 0.607 J/cm^2^. Briefly, the bacterial samples were treated with PAT using various photosensitizer formulations accompanied with led right irradiation for 15 minutes and then incubated on Petrifilm™ growth count plates to enable the counting of colony forming units (CFU). The concentration of photosensitizer as well as the concentration of EDTA used in the formulations for inactivation experiments were determined from optimum conditions determined earlier^53^.

### Determination of photosensitization mechanism

L-histidine and sodium azide were used as specific quenchers of singlet oxygen (^1^O_2_) produced during irradiation of TBO with light^49^. Combination of PAT with a known quencher allows us to monitor will affect the liberated ROS and cause changes in bacterial inhibition level^19^. In this study, the PAT of bacteria involving quenchers was carried out according to the procedure for bacterial inhibition studies described in a previous research^53^. Bacteria were grown overnight, and then resuspended in PBS to an optical density of 0.14, as compared to that of 0.12 (Corresponding to 10^8^ CFU/mL)^53^. This was necessary because the addition of the quencher would change the concentration of bacterial inoculum. To keep it unchanged from the previous case where only photosensitizer formulations were being added to the bacterial dispersions, a slightly higher inoculum density was used. In all the experiments involving the quencher, 400 µL of bacterial inoculum was added to each well of a 48-well tissue culture plate (Thermo Scientific). Following this, photosensitizer formulation of a volume of 50 µL was added to each well. Control wells were inoculated with sterile phosphate buffered saline. Following the addition of photosensitizer, 50 µL of the desired quencher with the appropriate concentration was added to the bacterial inocula. The samples were then irradiated with light, with control groups being incubated in the dark as well. Following light irradiation for 15 minutes, the total contents of each well were transferred to microcentrifuge tubes. After centrifugation, the supernatant (Containing the photosensitizer and the quencher) was discarded. The bacterial pellet in each microcentrifuge tube was resuspended in 500 µL of necessary medium, depending on the bacterial strain (Nutrient broth for *P*. *aeruginosa* and *S*. *aureus*). This was necessary to avoid the growth stimulating/inhibiting effects of some of the quenchers. The only effect aimed at investigating was the effect of the quenchers on the ROS generated during light irradiation, which could lead to changes in bacterial inhibition. Finally, 10 µL of bacterial suspension from each microcentrifuge tube was transferred to the wells of a 96-well plate already containing 90 µL of required growth medium (Basic Life Biosciences, Taiwan). The optical density was monitored at a wavelength of 430 nm each hour for a total duration of 4 hours. Finally, inhibitions were calculated as described using Eq. 1.

1 $${\rm{Inhibition}}\,( \% )=(1-\frac{{{\rm{OD}}}_{{\rm{sample}}4{\rm{hr}}-0{\rm{hr}}}}{{{\rm{OD}}}_{{\rm{control}}4{\rm{hr}}-0{\rm{hr}}}})\times 100$$

### Qualitative comparison of singlet oxygen generated from photosensitizer formulations

Singlet oxygen (^1^O_2_) is an important species generated during the photosensitization process. To monitor the generation of singlet oxygen, 1, 3-DPBF was used as a specific reactant. 1, 3-DPBF is highly specific toward singlet oxygen. In the presence of singlet oxygen, it undergoes a reaction to form a new product. Therefore, the decrease in quantity of 1, 3-DPBF is directly proportional to the amount of singlet oxygen present in the reaction environment. The absorbance of 1, 3-DPBF at 411 nm can provide a qualitative estimation of singlet oxygen generation during the photosensitization process^51^.

### Determination of amount of singlet oxygen (^1^O_2_) after PAT

A stock solution of 1000 µg/mL 1, 3-DPBF was prepared in HPLC-grade methanol and stored in the dark at 4 degrees. The singlet oxygen species were assayed after 1, 3-DPBF was freshly diluted in methanol to the concentration of 25 µg/mL. To quantify production of singlet oxygen, photosensitizer formulations consisting of TBO in water and lipid nanoparticle-encapsulated TBO were produced in different concentrations from the stock solution. Hundred-microliter aliquots of the photosensitizer formulations were transferred to the wells of a 96-well plate. Then, 100 µL of 1, 3-DPBF solution was added to each of the wells in the 96-well plate. The plate was irradiated with red light for 15 minutes. A control was also maintained in which the photosensitizer formulations were not irradiated with light. Finally, the levels of 1, 3-DPBF in each of the wells were monitored by measuring the absorbance at the wavelength of 411 nm. These absorbance levels were used to calculate a quantitative estimate of the amount of singlet oxygen liberated after the light irradiation process. A lower level of 1, 3-DPBF remaining in the wells after light irradiation indicated that a higher amount of singlet oxygen had been generated by the photosensitizer.

### Evaluation of singlet oxygen generation kinetics

Similar concentration of 1, 3-DPBF was used to monitor the kinetics of the singlet oxygen generation during the light irradiation process. Hundred-microliter amounts of photosensitizer formulations, consisting of TBO in water and lipid nanoparticle-encapsulated TBO, were inoculated in concentrations ranging from 5 to 25 µg/mL in a 96-well plate. Then, 100 µL of 25 µg/mL 1, 3-DPBF was added to the well containing photosensitizer. Following this step, light irradiation was done for different time durations. The amount of 1, 3-DPBF was monitored at 411 nm every 30 seconds for a total duration of 180 seconds. These levels were used to obtain an estimate about the time-dependent production of singlet oxygen after the photosensitizer was subjected to light irradiation. The rate of singlet oxygen production was proportional to the rate of decrease of 1, 3-DPBF absorbance at 411 nm as a function of irradiation time.

### Uptake of photosensitizer in bacterial cells

A spectroscopic method was used to determine the uptake of the TBO in water and lipid nanoparticle-encapsulated TBO formulations^46^. Briefly, bacterial strains were grown overnight and aliquots of 4.5 mL were moved to 15 mL centrifuge tubes. They were inoculated with 0.5 mL of either TBO in water or lipid nanoparticle-encapsulated TBO, each with a final concentration of 15–20 ppm. The tubes were then incubated in dark at 37 degrees centigrade in a shaker-incubator. After 15 minutes of incubation combined with shaking at 100 rpm, each of the tubes was centrifuged at 10,000 rpm for 5 minutes. The supernatant was discarded, and the pellet was washed gently with PBS once to remove loosely bound photosensitizer molecules. The pellet was re-dissolved in 5 mL of solution containing 1% sodium dodecyl sulfate with 0.1 M NaOH solution. The tubes were stored at 4 degrees centigrade for 16–18 hours. They were then shaken for 30 minutes on an orbital. Next, 100 µL amounts of solution from each tube were loaded to the wells of a 96-well plate. Absorbance readings were taken at 630 nm using a spectrophotometer, and these values were used to calculate the concentration of the photosensitizer present in the solution by correlating with the standard curve of photosensitizer obtained earlier in the same solution. To calculate the exact number of photosensitizer molecules undergoing uptake by each bacterial cell, the CFU of the bacterial solution was also simultaneously determined using Petrifilm aerobic growth count plates.

### Agarose gel electrophoresis assay

Damage to bacterial DNA was assessed with agarose gel electrophoresis assay. Bacterial samples were treated with PAT using TBO in water and lipid nanoparticle-encapsulated TBO. DNA was then extracted from the control bacterial samples and from those treated with PAT. The DNA extraction method was modified from the procedures of previous researchers^54^. Briefly, the bacterial samples were treated with PAT using TBO formulations, and then DNA was extracted from the bacterial cells. Following purification procedures, these extracted DNA samples were run on the lanes of an agarose gel and analyzed for any changes.

### Bacterial DNA exudation after PAT

If cellular membrane integrity is compromised, various intracellular components can leak into the surrounding medium. One of the methods for monitoring bacterial DNA is by the absorbance at 260 nm^43^. Previous researchers have used this method to study damage occurring to bacterial cells after PAT^46^. Bacteria were treated with PAT using various concentrations of TBO in water and TBO in lipid nanoparticles, according to the protocol described in the preceding sections. Control samples included bacterial inoculum treated with PAT and incubated in dark. Contents from each well of the 48-well plate were then transferred to microcentrifuge tubes. They were then subjected to centrifugation at 11,000 rpm for 5 minutes, and the supernatant was collected in another set of tubes. This was done to discard the pellet, as taking the absorbance of the solutions containing the bacterial cells can lead to some interference in the assay. Finally, 100 µL aliquots of the cell-free supernatants were transferred to the wells of a 96-well plate, and absorbance readings were taken at a wavelength of 260 nm. Each time, a blank reading consisting of unloaded wells in the multi-well plate was also taken and subtracted from the readings of the other samples.

### Fluorescence microscopy and scanning electron microscopy

For studying *S*. *aureus* SA 113 biofilm viability after PAT, staining was done using Hoechst dye (bisBenzimide 33342, Sigma Aldrich) and propidium iodide (Sigma Aldrich) dyes. Bacteria were grown overnight in a tube containing 3 mL of tryptic soy broth supplemented with 0.5% glucose. After resuspending in PBS to an OD_600_ of 0.12, 50 µL was added to the wells of a Thermo Scientific Nunclon 48-well plate. The plate was incubated at 37 °C for 3 days (without shaking), with medium change on second day. On the third day, PAT was done on the biofilm by adding 100 µL of TBO formulations and irradiating with red light at 15 cm distance for 15 minutes. After PAT, 100 µL of the desired concentration of the Hoechst dye (10 ppm) and propidium iodide (1 ppm) dyes were added. Incubation was done in dark for 15 minutes at 37 °C. To remove loosely-bound dye, biofilm-containing wells were washed gently with PBS after removing the dye solution. Analysis was done under a Leica DM IL microscope (Leica AG, Germany) with 10x objective and appropriate filters. Images were taken by a camera coupled to the microscope (CoolSNAP cf CCD camera; Photometrics, Arizona) and controlled by MetaVue imaging software (Molecular Devices, California).

Thermanox® plastic cover slips of 13 mm diameter from NUNC (Rochester, NY USA) were used. The cover slips were added to the bottom of the wells of a 48-well plate. *S*. *aureus* SA 113 bacterial biofilms were grown on the plastic cover slips and treated with PAT according to the procedure described in the preceding section. After PAT, the biofilms on the plastic cover slips were fixed according to a protocol described in a previous research^53^. Examination was finally done under a SEC Mini SEM SNE-4500M SEM (MSITECH).

### Statistical methods

Data are reported as means ± standard deviations (n = 3). ANOVA and post-hoc two-tailed t-tests inbuilt in MS Excel were used for evaluating the differences between the means of results for control groups and the groups treated with the photosensitizer. Unless specifically mentioned, *P* values of less than 0.05 were considered significant.

## Electronic supplementary material

Supplementary information
