# Supplementary material for: Photosensitizer in lipid nanoparticle: a nano-scaled approach to antibacterial function
Source: Sci Rep. 2017 Aug 11;7:7892. doi: 10.1038/s41598-017-07444-w (PMC5554217; doi:10.1038/s41598-017-07444-w)
Supplement: Supplementary file 1 — Supplementary information [file 41598_2017_7444_MOESM1_ESM.pdf]

Supporting Information

Photosensitizer in lipid nanoparticle: a nano-scaled approach to antibacterial function

Bishakh Rout [a], Chi-Hsien Liu [a,b,c,d*], Wei-Chi Wu [d,e]

[a] Graduate Institute of Biochemical and Biomedical Engineering, Chang Gung University, 259, Wen-Hwa First Road, Kwei-Shan, Tao-Yuan 333, Taiwan

[b] Research Center for Chinese Herbal Medicine and Research Center for Food and Cosmetic Safety, College of Human Ecology, Chang Gung University of Science and Technology, 261, Wen-Hwa First Road, Taoyuan, Taiwan

[c] Department of Chemical Engineering, Ming Chi University of Technology, 84, Gung-Juan Road, New Taipei City, Taiwan

[d] Department of Ophthalmology, Chang Gung Memorial Hospital, 5, Fu-Hsing Street, Taoyuan, Taiwan

[e] College of Medicine, Chang Gung University, 259, Wen-Hwa First Road, Taoyuan, Taiwan

Corresponding author's E mail: CHL@mail.cgu.edu.tw

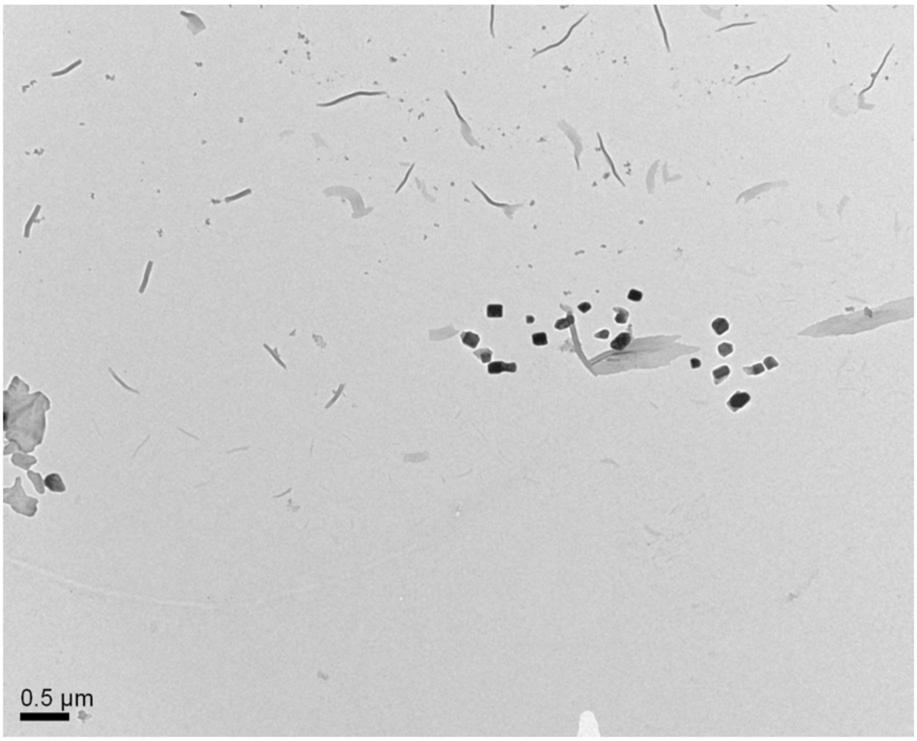

Fig. S1 (a) TEM image of TBO dispersed in water - At 15000× magnification (Scale bar = 0.5 μm), it shows aggregation and non-uniform size. Samples were diluted appropriately and loaded onto copper grids. After drying overnight, TEM images were acquired.

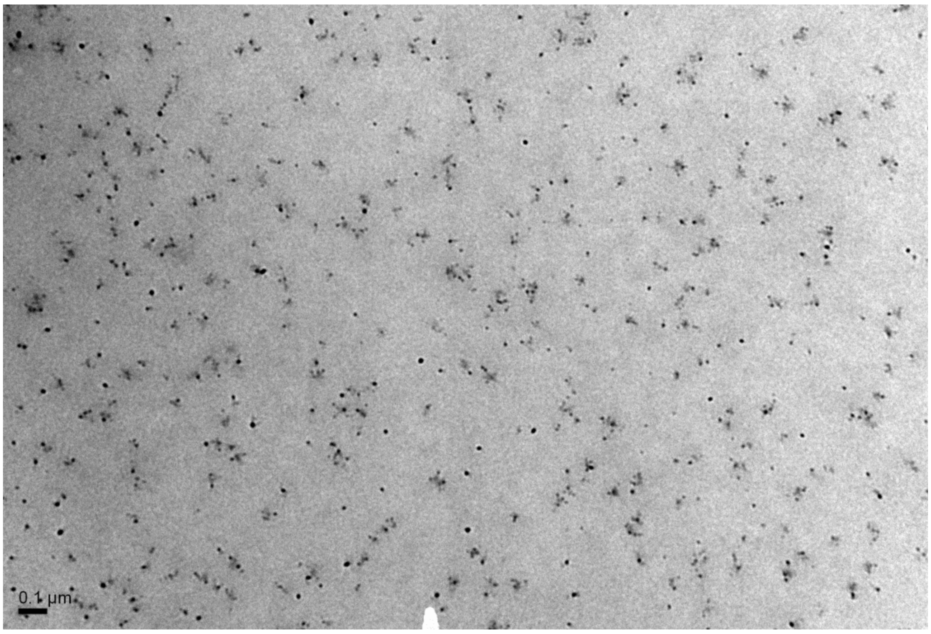

Fig. S1 (b) TEM image of TBO in lipid nanoparticles (TLN)- At 60000× magnification (Scale bar = 0.1 μm), it shows a smaller particle size and uniform size distribution. Samples were diluted appropriately and loaded onto copper grids. After drying overnight, TEM images were acquired.

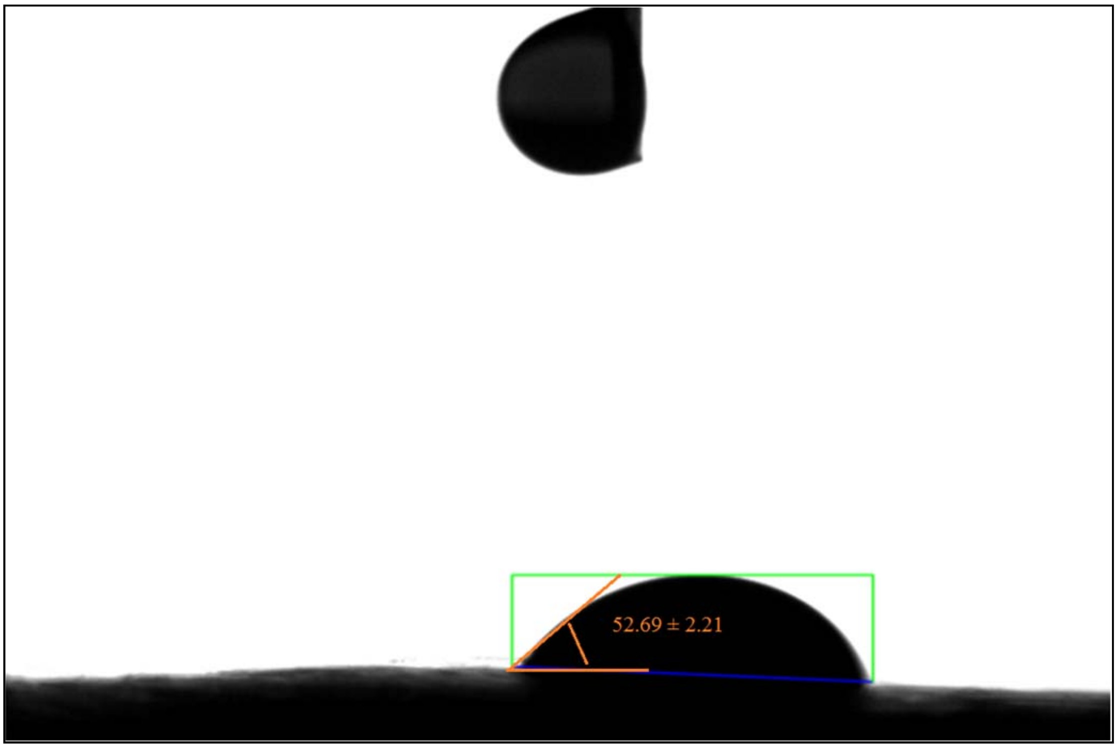

Fig. S2 (a) Contact angle measurements of TBO in water on porcine skin. Image was shown for representation only. The measurements in Table 1 were taken as an average of both the left and right angles of the droplet, and counted from three experimental runs using three droplets

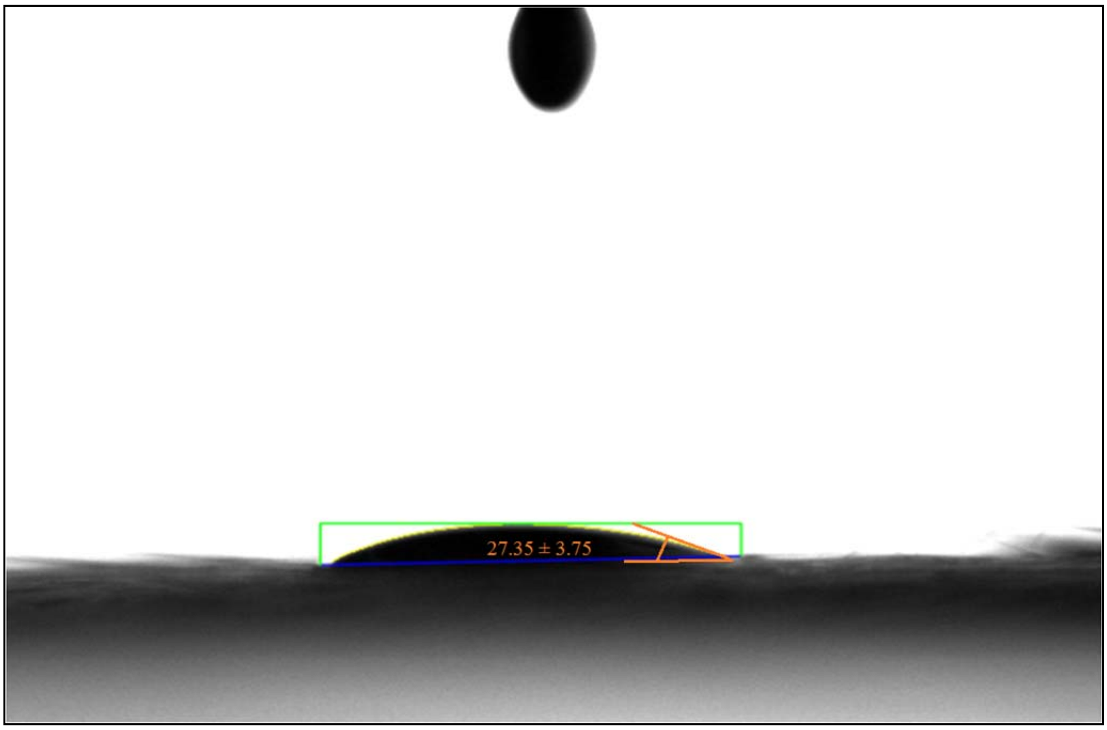

Fig S2(b) Contact angle measurements of TBO in lipid nanoparticles on porcine skin. Image was shown for representation only. The measurements in Table 1 were taken as an average of both the left and right angles of the droplet, and counted from three experimental runs using three droplets

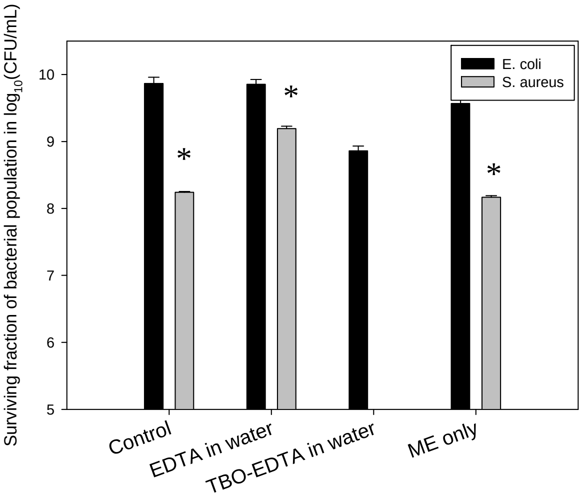

Fig. S3 Effect of various formulations on inhibition of (a) S. aureus and (b) E. coli - Neither EDTA alone nor ME alone without TBO had any effect on the bacteria. TBO combined with EDTA also did not have much effect on the bacteria. Significant differences have been denoted at a probability value < 0.05 (*).

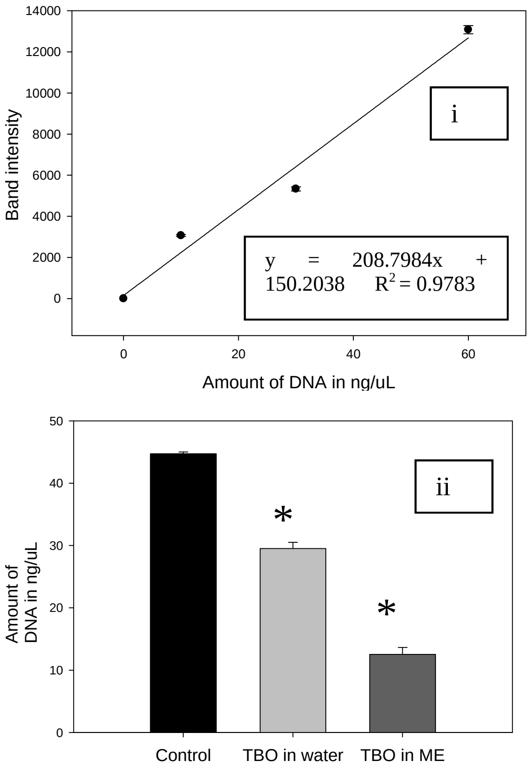

Fig. S4 (a) (i) Standard curve for band intensity - DNA amount in *P. aeruginosa* and (ii) changes in DNA levels in *P. aeruginosa* after treatment with PDT using TBO in water and TBO in lipid nanoparticles (TLN). Significant differences have been denoted at a probability value < 0.05 (*).

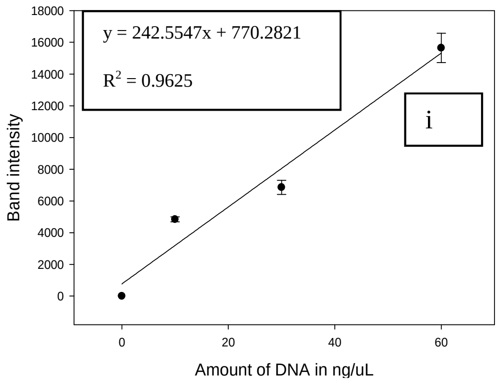

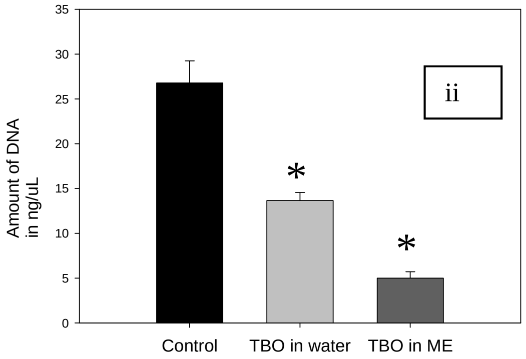

Fig. S4 (b) (i) Standard curve for band intensity - DNA amount in *E. coli* and (ii) changes in DNA levels in *E. coli* after treatment with PDT using TBO in water and TBO in lipid nanoparticles (TLN). Significant differences have been denoted at a probability value < 0.05 (*).

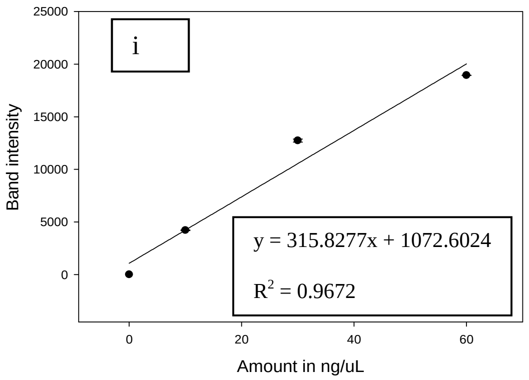

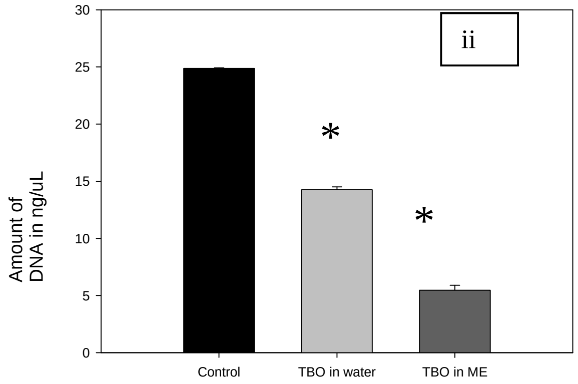

Fig. S4 (c) (i) Standard curve for band intensity - DNA amount in *S. aureus* and (ii) changes in DNA levels in *S. aureus* after treatment with PDT using TBO in water and TBO in lipid nanoparticles (TLN). Significant differences have been denoted at a probability value < 0.05 (*).

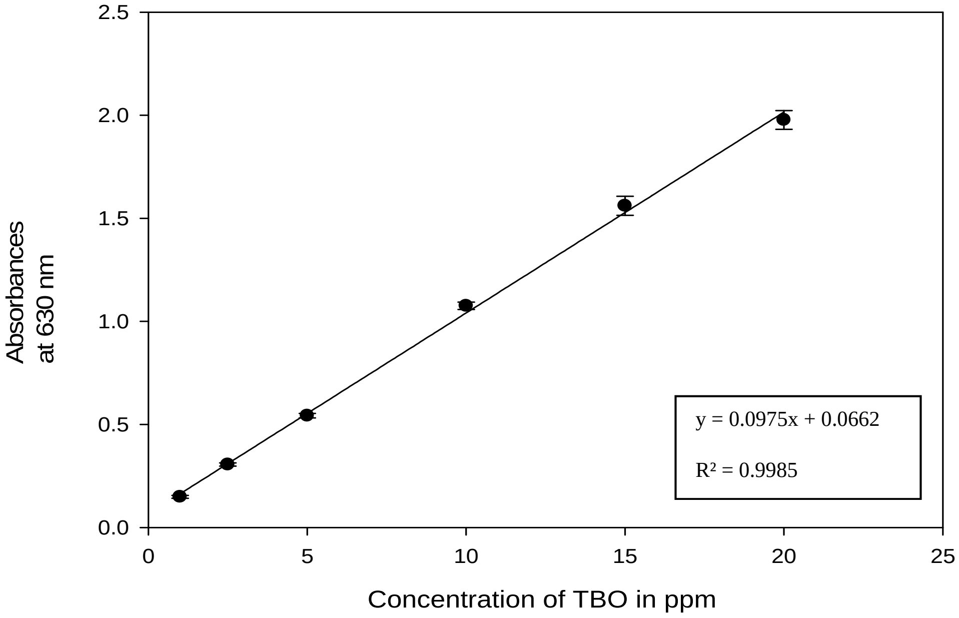

Fig. S5 (a) Standard curve of absorbances at 630 nm for TBO in water - 100 µL aliquots of TBO in water at various concentrations were loaded into the wells (n=3) of a 96-well plate and absorbances monitored at a wavelength of 630 nm on a microplate reader (Synergy, Biotek, Hong Kong)

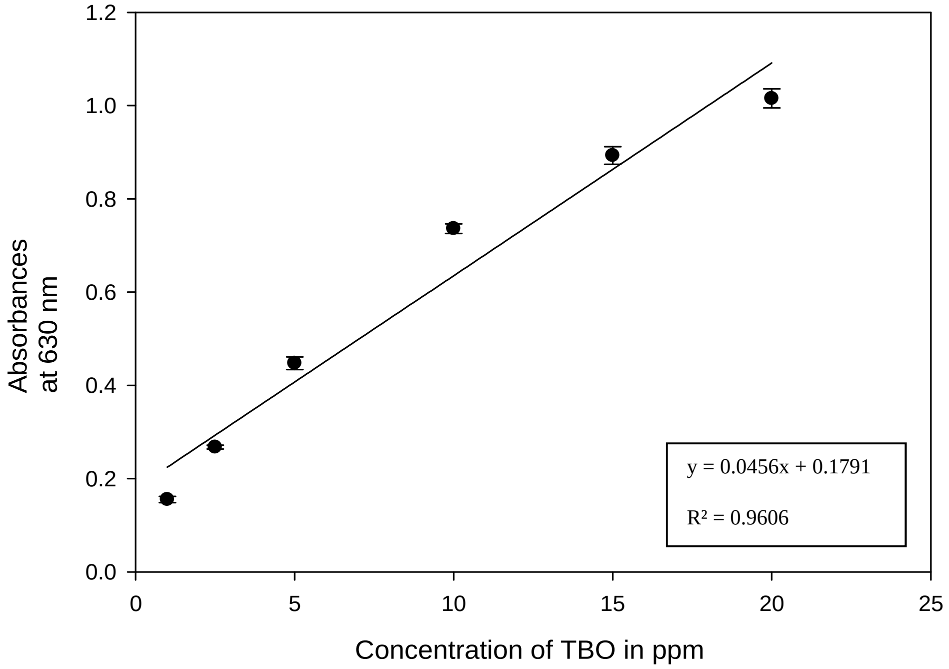

Fig. S5 (b) Standard curve of absorbances at 630 nm for TBO in lipid nanoparticles- 100 µL aliquots of TBO in lipid nanoparticlesat various concentrations were loaded into the wells of a 96-well plate (n=3) and absorbances monitored at a wavelength of 630 nm on a microplate reader (Synergy, Biotek, Hong Kong)

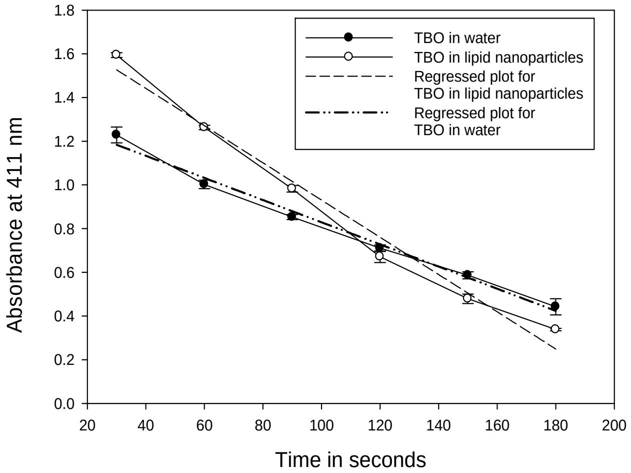

Fig. S6 (a) Kinetics of singlet oxygen generation with photosensitiser concentration of 1 ppm - Absorbances of 1,3-DPBF were monitored at 411 nm during light irradiation process of TBO every 30 seconds, for a total period of 180 seconds (n=3)

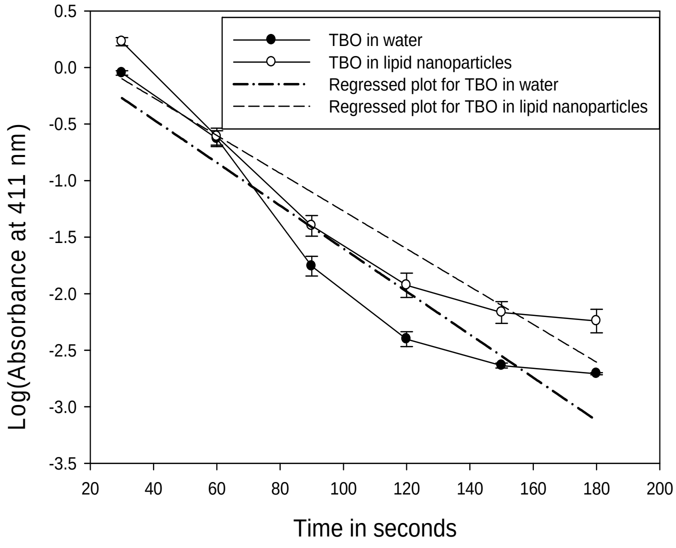

Fig. S6 (b) Kinetics of singlet oxygen generation with photosensitiser concentration of 5 ppm - Absorbances of 1,3-DPBF were monitored at 411 nm during light irradiation process of TBO every 30 seconds, for a total period of 180 seconds (n=3)

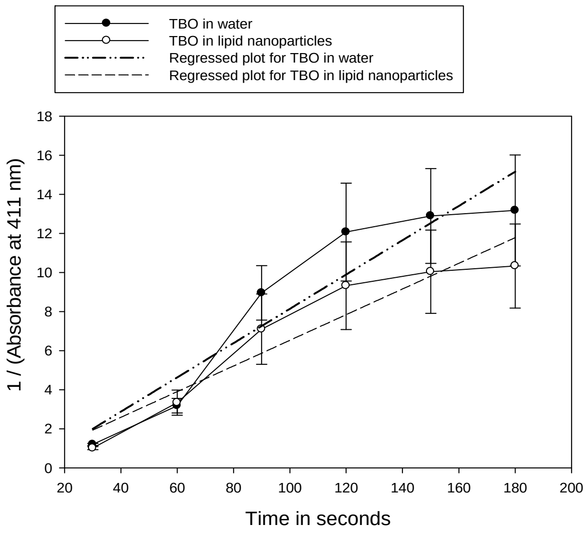

Fig. S6 (c) Kinetics of singlet oxygen generation with photosensitiser concentration of 10 ppm - Absorbances of 1,3-DPBF were monitored at 411 nm during light irradiation process of TBO every 30 seconds, for a total period of 180 seconds (n=3)

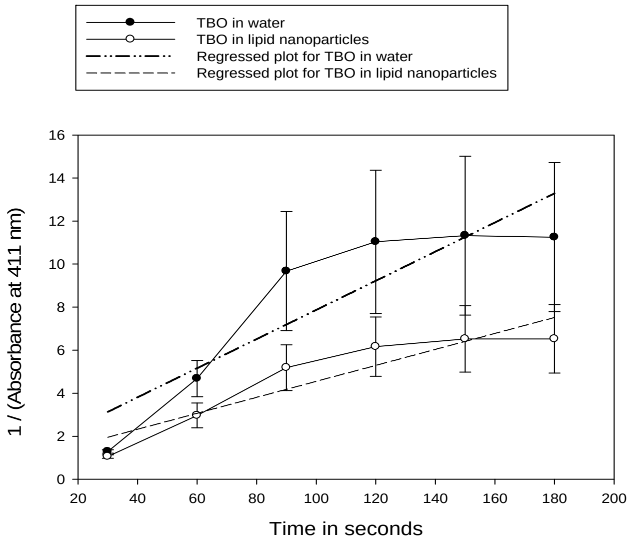

Fig. S6 (d) Kinetics of singlet oxygen generation with photosensitiser concentration of 25 ppm - Absorbances of 1,3-DPBF were monitored at 411 nm during light irradiation process of TBO every 30 seconds, for a total period of 180 seconds (n=3)

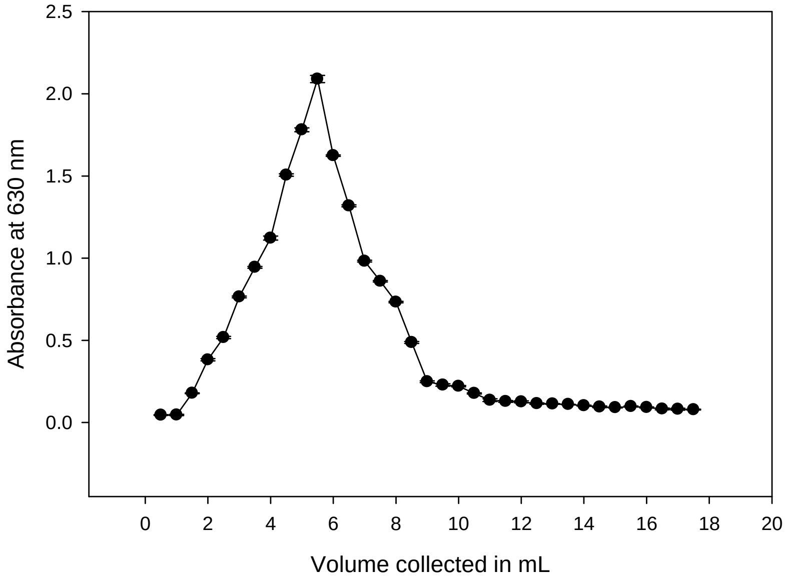

Fig. S7 Elution profile of TBO in lipid nanoparticles using size exclusion chromatography - The figure shows the absorbance spectrum obtained after the size exclusion chromatography experiment. The error bars represent the standard deviation of the measurements resulting from three separate experiments carried out under the same conditions.

For measuring the encapsulation efficiency of TBO in the ME, size exclusion chromatography was carried out. Sephadex G-50 Superfine (G.E., USA) was used for the encapsulation efficiency measurements. The gel was prepared by dispersing the supplied powder in PBS according to the manufacturer's instructions. The gel was then filled to a pre-determined height in a tube.

The TBO-loaded ME was then loaded onto the gel column, and the diffusing liquid was collected from the bottom of the gel column in micro-centrifuge tubes. An amount of 0.5 mL was collected in each micro-centrifuge tube. After the diffusion of the whole inoculated sample was completed, the samples were added to the wells of a 96-well micro titer plate (Basic Life BioSciences, Taiwan) for quantification by using the wavelength of 430 nm in a microplate reader (Synergy HT, BioTek, Hong Kong). Encapsulation efficiency (EE) was calculated by using the following equation.

$$\text{Encapsulation efficiency (EE)} = \left(\frac{Actual\ amount\ of\ drug\ loaded\ in\ microemulsion}{Theoretical\ amount\ of\ drug\ loaded\ in\ microemulsion}\right) \times 100$$

[Eq. S1]

According to the size exclusion chromatography experiments, the encapsulation efficiency of TBO in lipid nanoparticles was high at 83.01 %.

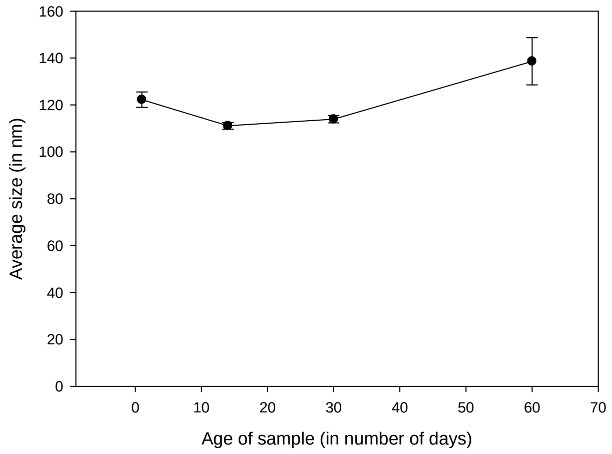

Fig. S8 Effect of storage duration on the average particle size of TBO in lipid nanoparticles- No significant change occurred, thereby proving that aggregation was prevented

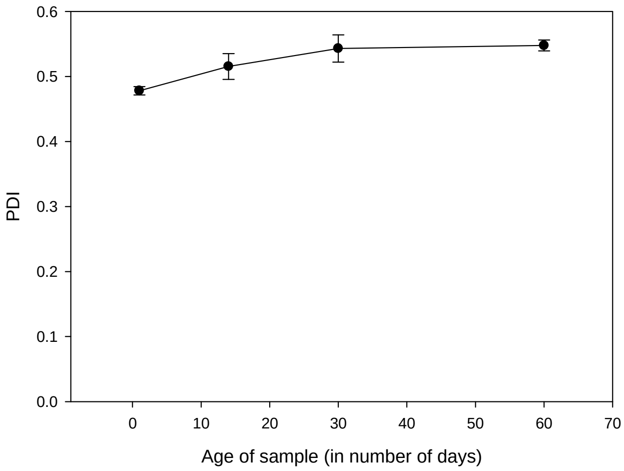

Fig. S9 Effect of storage duration on the PDI of TBO in lipid nanoparticles- No significant change occurred, thereby proving that aggregation was prevented.

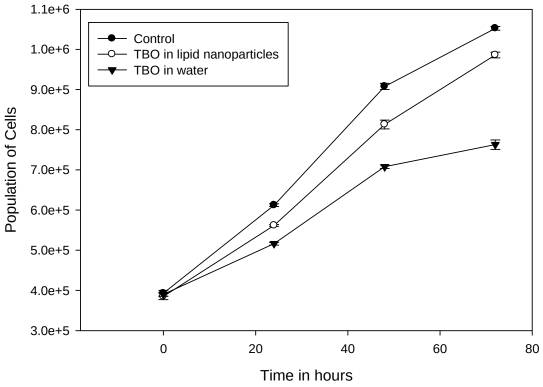

Fig. S10 Results of the cell toxicity assay carried out on the HB-9645 suspension cells - The toxicity to the cells was decreased after the usage of lipid nanoparticles for encapsulating TBO. The control group was just maintained in RPMI-1640 medium. Population of the cells was monitored using a Beckman-Coulter MS3 Multisizer.

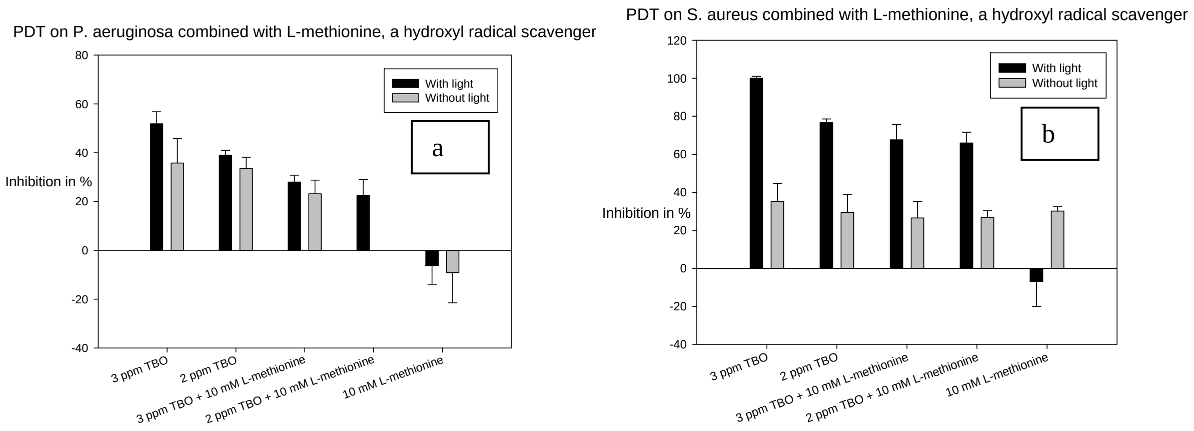

Fig. S11 Effect of L-methionine combined with TBO in water on inhibition of (a) *Pseudomonas aeruginosa* and (b) *Staphylococcus aureus* - Inhibition decreased in both the cases after combination with L-methionine

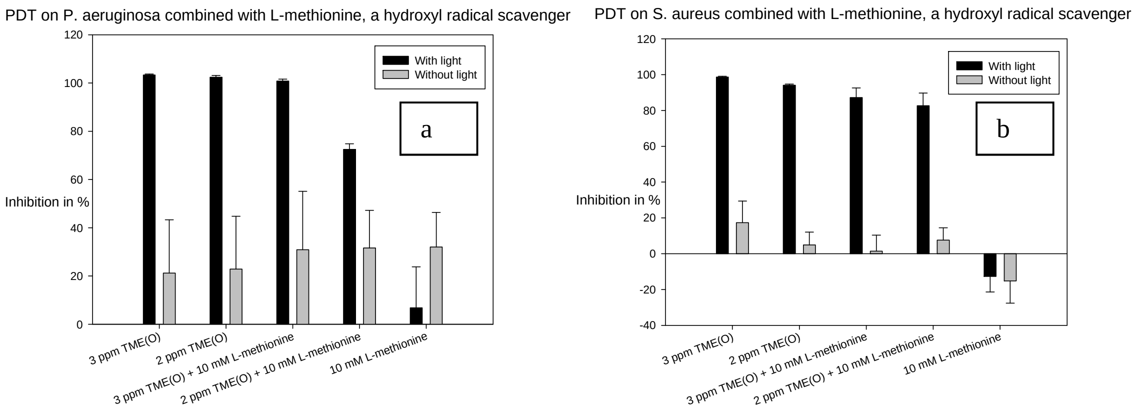

Fig. S12 Effect of L-methionine combined with TBO in lipid nanoparticles on inhibition of (c) *Pseudomonas aeruginosa* and (d) *Staphylococcus aureus* - Inhibition decreased slightly in both the cases after combination with L-methionine
